# Supplementary material for: Optimal control of multiple myeloma assuming drug resistance and off-target effects
Source: PLoS Comput Biol. 2025 Aug 11;21(8):e1012225. doi: 10.1371/journal.pcbi.1012225 (PMC12364355; doi:10.1371/journal.pcbi.1012225)
Supplement: S1 Text — Detailed numerical methods used, including required mathematical derivations. This complements the methods described in the main text. (PDF) [file pcbi.1012225.s001.pdf]

S1 Text - Methods

James G. Lefevre[1,2*], Brodie A.J. Lawson[2,3], Pamela M. Burrage[2,3], Diane M. Donovan[1,2], Kevin Burrage[2,3,4]

1 School of Mathematics and Physics, The University of Queensland, Brisbane, Australia
2 ARC Centre of Excellence, Plant Success in Nature and Agriculture
3 School of Mathematical Sciences, Queensland University of Technology, Brisbane, Australia
4 Department of Computer Science, University of Oxford, Oxford, UK

* j.lefevre@uq.edu.au

A Application of Pontryagin's maximum principle

We begin by outlining the application of Pontryagin's maximum (minimum) principle to solve an optimal control problem as specified in Eqs (1–3) in the main text. Methods broadly follow [1] except for modifications to the convergence algorithm for the linear cost function and to the method of obtaining equilibrium solutions.

Consider a boundary value problem of the form given by Eqs (1) and (2), where $\mathbf{x}(t) = (x_1(t), x_2(t), \ldots, x_n(t))$ is a vector of state variables and $u(t)$ is the control. The objective is to choose $u(t)$ to minimise a cost function of the form (3) over a time window $[t_0, t_f]$. We introduce a vector of costate variables $\boldsymbol{\lambda}(t) = (\lambda_1(t), \lambda_2(t), \ldots, \lambda_n(t))$ and define a Hamiltonian

$$H(t) = \mathcal{L}(t) + \boldsymbol{\lambda}(t)\mathbf{f}(t).$$

The costate variables can be obtained from the necessary conditions

$$\frac{d\boldsymbol{\lambda}}{dt} = -\frac{\partial H}{\partial \mathbf{x}} = -\left(\frac{\partial \mathcal{L}}{\partial \mathbf{x}} + \boldsymbol{\lambda}\frac{\partial \mathbf{f}}{\partial \mathbf{x}}\right) \tag{A}$$

and the transversality condition

$$\boldsymbol{\lambda}(t_f) = \left.\frac{\partial \phi}{\partial \mathbf{x}}\right|_{t=t_f}. \tag{B}$$

Pontryagin's maximum (minimum) principle [2] states that the cost function is minimised when the control, together with the corresponding state and costate, minimise $H(t)$ for all $t \in [t_0, t_f]$. In general this is not directly solvable, as $\mathbf{x}$ and $\boldsymbol{\lambda}$ must be obtained numerically for a given u. We use the following approach, where at each step $t \in [t_0, t_f]$:

Algorithm 1

1. Select an initial value for $u(t)$.
2. Solve the boundary value problem given by the state Eqs (1) and (2) for $\mathbf{x}(t)$.
3. Solve the boundary value problem given by the costate Eqs (A) and (B) for $\boldsymbol{\lambda}(t)$.

4. Find $u^*(t)$ which minimises $H(t)$ for the given $\mathbf{x}(t)$ and $\boldsymbol{\lambda}(t)$.
5. Update $u(t)$ to a new value $u_{new}(t)$ based on a combination of the current value $u_{old}(t)$ and $u^*(t)$.
6. Check the specified convergence condition; if not met, go to step 2.

We use the initial control value $u(t) = 0$ in all cases. The state equations $\mathbf{f}(\mathbf{x}, u)$ are given by Eqs (4–6) in the main text, with $\mathbf{x} = (A, P, N)$. In all cases the initial state is a stable equilibrium with cancer present and no control (see below). We solve the boundary value problems using the fourth order Runge-Kutta method and time step 0.001. In step 3, the boundary value is specified at time t_f, so the solution is obtained working backwards in time. The details of the costate equations and of steps 4, 5 and 6 depend on the cost function and the corresponding optimal control form, as discussed in the main text Introduction. We consider the two cases separately below. We use the convergence condition $|u_{new} - u_{old}|/|u_{new}| < 10^{-3}$.

B Control for quadratic cost function

We first consider the quadratic cost function $\mathcal{L} = u^2 + (P + N)^2$ (Eq (8) in the main text), which will typically correspond to continuous optimal control solutions. Using Eq (A) with $\mathbf{f} = \left(\frac{dA}{dt}, \frac{dP}{dt}, \frac{dN}{dt}\right)$ given by Eqs (4–6) in the main text, we obtain the costate equations

$$\begin{aligned}
\frac{d\lambda_1}{dt} &= \lambda_1 \left(-\rho_A(1 - 2A - P - N) + \mu_A + \mu_{Au}u\right) + \lambda_2\rho_P P + \lambda_3\rho_N N \\
\frac{d\lambda_2}{dt} &= -2(P + N) + \lambda_1\rho_A A + \lambda_3 \left(\rho_N N - \delta_P - \delta_{Pu}u - \frac{\alpha N}{(\gamma + P + N)^2}\right) \\
&\quad +\lambda_2 \left(-\rho_P(1 - A - 2P - N) + \delta_P + \delta_{Pu}u + \mu_P + \mu_{Pu}u + \frac{\alpha(\gamma + N)}{(\gamma + P + N)^2}\right) \\
\frac{d\lambda_3}{dt} &= -2(P + N) + \lambda_1\rho_A A + \lambda_2 \left(\rho_P P - \delta_N - \frac{\alpha P}{(\gamma + P + N)^2}\right) \\
&\quad +\lambda_3 \left(-\rho_N(1 - A - P - 2N) + \delta_N + \mu_N + \frac{\alpha(\gamma + P)}{(\gamma + P + N)^2}\right).
\end{aligned}$$

The end-state cost ϕ in the cost function (Eq (3) in the main text) is set to zero, so the boundary condition (B) gives $\boldsymbol{\lambda}(t_f) = 0$. We next find $u^*(t)$ that minimises H for all $t \in [t_0, t_f]$. Note that

$$\psi = \frac{\partial H}{\partial u} = 2u - \lambda_1\mu_{Au}A - \lambda_2\delta_{Pu}P - \lambda_2\mu_{Pu}P + \lambda_3\delta_{Pu}P.$$

Setting $\psi(t) = 0$ gives

$$u^* = (\lambda_1\mu_{Au}A + \lambda_2\delta_{Pu}P + \lambda_2\mu_{Pu}P - \lambda_3\delta_{Pu}P)/2.$$

Using u^* as the updated value of u will not in general allow for convergence, and so the updated value of u is then taken to be a linear combination of the current value and u^*,

$$u_{new}(t) = \omega u_{old} + (1 - \omega)u^*.$$

The parameter $\omega \in [0, 1)$ can be increased as required to achieve convergence; we used $\omega = 0.9$ in all cases, with convergence in less than 100 steps.

C Control for linear cost function

We now consider the linear cost function $\mathcal{L} = u + P + N$ (Eq (7) in the main text). The costate equations differ only slightly from the continuous control case, with the term $-2(P+N)$ replaced by -1 in the equations for $\frac{d\lambda_2}{dt}$ and $\frac{d\lambda_3}{dt}$. The costate boundary condition is again $\boldsymbol{\lambda}(t_f) = 0$. However, a substantially different approach is required for the optimality condition. Note that

$$\psi = \frac{\partial H}{\partial u} = 1 - \lambda_1 \mu_{Au} A - \lambda_2 \delta_{Pu} P - \lambda_2 \mu_{Pu} P + \lambda_3 \delta_{Pu} P.$$

Since $\psi(t)$ is not a function of u, the natural approach to minimising H is to increase u where $\psi(t) < 0$, and decrease u where $\psi(t) > 0$, while limiting the change in u in each step to allow convergence given the indirect effect of u on H via $\mathbf{x}$ and $\boldsymbol{\lambda}$. However, this will generally result in u diverging towards $\pm\infty$ at some values of t, and so it is necessary to impose bounds $u_0 < u(t) < u_1$. Such bounds are typically consistent with the real world system being modelled. In our model, the drug level cannot be negative and there will be an upper bound on the safe and effective drug dose; we use the range $[u_0, u_1] = [0, 1]$. In the approach adopted from [1], we set $u^*(t) = u_0$ for $\psi(t) > 0$, and $u^*(t) = u_1$ for $\psi(t) < 0$. We then set $u_{new}(t) = \omega u_{old} + (1-\omega)u^*$, as for the continuous control. In some cases we found that this algorithm converged to a solution in which $u(t)$ is equal to either 0 or 1 for each $t \in [t_0, t_f]$, the expected form of a bang-bang control. But note that while $u^*(t)$ has this form at each time step by definition, the update method using a weighted average means that $u(t)$ does not have this form at each step of the iteration. In fact, we found that for some parameter cases we could not meet the formal convergence criterion for any choice of ω, yet the control appeared to approximately converge to a form in which $u(t)$ attains a value intermediate between 0 and 1 for a range of t. The algorithm given above cannot achieve convergence to such a control, since $u^*(t)$ will be equal to 0 or 1 for any given iteration and value of t. In order to achieve convergence to optimal control solutions of this type, we adopted the following convergence strategy:

$$\begin{aligned} u^*(t) &= u_{old}(t) - \omega\psi(t), \\ u_{new}(t) &= \max(u_0, \min(u_1, u^*(t))). \end{aligned}$$

The convergence parameter $\omega > 0$ is not comparable to the parameter in the previous method. Lower values give slower but more reliable convergence. We used $\omega \in \{0.02, 0.01, 0.005\}$. Convergence speed was found to be substantially lower than for continuous controls even for simple bang-bang controls using the original algorithm. For the modified algorithm we added an additional secondary convergence condition, calculated every 1000 iterations: $|u_t - u_{t-1000}|/|u_t| < 10^{-2}$. For obtaining controls including periods of intermediate control values using the modified method, convergence speed was substantially slower again, with up to 10000 iterations required for convergence, and up to 60000 iterations were required for more complex control solutions.

D Accounting for finite time horizon

A limitation of the optimal control methodology used is that results are valid only for the specified time window, while we are typically interested in the cost of treatment over an indefinite period. This includes cases where ongoing treatment is required. If the model parameters do not permit permanent control of the cancer, the optimal control may include an artefact late in the time window caused by the artificial

end-point. For this reason all optimal controls were obtained using a time window $[0, 200]$ (unless otherwise specified), and only the period $[0, 100]$ or $[0, 50]$ was shown. All costs and other statistics shown or plotted were recalculated on this interval.

For the primary analysis we assume that if the computed optimal control reaches an apparent steady state within the period analysed, then this apparent steady state represents the infinite horizon time-averaged cost optimal treatment regime. We also make the related assumption that such a control solution is not subject to any finite time window distortion. We test these assumptions in a supplementary analysis (Section D in S2 Text) using two approaches.

Firstly, for each parameter selection considered we directly compute the infinite time horizon, time-averaged optimal treatment solution and compare it to the steady state. In every case analysed with $\alpha > 0$, the cancer and treatment levels appear to go to zero within the time window, and we need only verify that the $N = P = 0$ equilibrium is an fact stable with $u = 0$ (Section E). In the remaining cases, where $\alpha = 0$, we can obtain the unique stable steady state corresponding to any fixed value of u by making the parameter substitution $\mu'_A = \mu_A + u\mu_{Au}$, $\delta'_P = \delta_P + u\delta_{Pu}$, $\mu'_P = \mu_P + u\mu_{Pu}$ and applying the method given in Section E.1. We then numerically calculate the value of u which, with its associated stable steady state, minimises the value of $\mathcal{L}$ given by Eq (8) (quadratic cost) or Eq (7) (linear cost) in the main text. We used the Matlab function fminbnd on $u \in [0, 1]$. Minimum costs obtained were all less than 1, so no lower cost is possible for $u > 1$.

E Equilibria

All optimal control calculations use an initial state $\mathbf{x}(t_0)$ in which cancer is present and the system is in stable equilibrium prior to application of the control. We do not have an exact expression for this steady state, so we first find the steady state solution with $N, P > 0$ for the restricted model with $\alpha = 0$ (no immune response). We then run the full model simulation, using the fourth order Runge-Kutta method and time step 0.001 as in the optimal control algorithm, until the absolute single time step change in each state variable is less than the minimum floating point difference (2^{-53}). We cannot prove uniqueness for this steady state, however we performed a numerical check for the default model parameters. We used a grid search with initial values $A, P, N \in \{0, 0.1, 0.2, \ldots, 1\}$, calculating the trajectories and determining that every trajectory converged towards either $(A, P, N) = (0.318, 0.381, 0.015)$ or $(A, P, N) = (0.423, 0, 0)$ (it is easily seen that no physical equilibria can exist for A, P or N greater than 1, except possibly when $N = P = 0$).

In the following we provide the required derivation for the steady states with $\alpha = 0$. We also show that the physically realisable steady states for this model conform to one of two cases: (1) there is exactly one steady state, which has $N = P = 0$ and is stable; (2) there are two steady states, one with $N = P = 0$ which is unstable, and one with $P, N > 0$ which is stable. In particular, this shows that the Michaelis-Menten immune response term is necessary in order to allow for cases in which cancer is present and persistent, but can be permanently controlled by a finite period of drug treatment. Note that in the neighbourhood of $P = N = 0$, the full model is equivalent to a model with $\alpha = 0$ and modified cancer exit rates $\mu'_P = \mu_P + \alpha/\gamma$ and $\mu'_N = \mu_N + \alpha/\gamma$. Using the method below we can thus determine exactly when the $N = P = 0$ equilibrium is stable. However, in the full model a stable $P = N = 0$ equilibrium does not exclude a stable equilibrium with $N, P > 0$, due to the reduction in the immune component of the exit rates as $P + N$ increases.

E.1 Equilibria with zero immune response

To obtain the steady states of our model with no control and in the absence of the immune response, we let $C = 1 - A - P - N$ and set $\alpha = u = \frac{dA}{dt} = \frac{dP}{dt} = \frac{dN}{dt} = 0$ in Eqs (4–6) in the main text to give

$$\beta_A + \rho_A AC - \mu_A A = 0 \tag{C}$$
$$\rho_P PC - \delta_P P + \delta_N N - \mu_P P = 0 \tag{D}$$
$$\rho_N NC + \delta_P P - \delta_N N - \mu_N N = 0. \tag{E}$$

In the following we assume that all parameters are positive. For physical (non-negative) N and P, we have $A \le 1 - C$. With Eq (C) this gives the condition $\rho_A C^2 - (\rho_A + \mu_A)C + \mu_A - \beta_A \ge 0$. The larger zero of the quadratic function is greater than 1 and hence non-physical, thus

$$C \le \frac{\rho_A + \mu_A}{2\rho_A} - \sqrt{\left(\frac{\rho_A - \mu_A}{2\rho_A}\right)^2 + \frac{\beta_A}{\rho_A}}. \tag{F}$$

For any C satisfying this constraint, we obtain A by rearranging Eq (C) to give $A = \beta_A/(\mu_A - \rho_A C)$. We always have a non-cancer steady state in which $P = N = 0$, where C is equal to the bound in Eq (F) and $A = 1 - C$. By Eqs (D) and (E), $N = 0 \iff P = 0$, so it remains only to consider the case $N > 0$ and $P > 0$. From Eqs (D) and (E) we have

$$\frac{P}{N} = \frac{\delta_N}{\delta_P + \mu_P - \rho_P C} = \frac{\delta_N + \mu_N - \rho_N C}{\delta_P}. \tag{G}$$

This gives a quadratic equation in C. The larger solution is greater than 1, and hence the only potentially physical solution is

$$C = \frac{\delta_P + \mu_P}{2\rho_P} + \frac{\delta_N + \mu_N}{2\rho_N} - \sqrt{\left(\frac{\delta_P + \mu_P}{2\rho_P} - \frac{\delta_N + \mu_N}{2\rho_N}\right)^2 + \frac{\delta_P \delta_N}{\rho_P \rho_N}}. \tag{H}$$

We see that there are two cases. There is always a non-cancerous steady state. If the expression for C in Eq (H) satisfies condition (F) without exact equality, and gives a positive value for the ratio P/N in Eq (G), then A can then be obtained from Eq (C), and the cancerous cells $N + P = 1 - A - C$ can be apportioned according to Eq (G) to give exactly one additional physical solution.

We next consider the stability of these equilibria. The Jacobian for the system when $\alpha = u = 0$ is

$$J = \begin{bmatrix} \rho_A(C - A) - \mu_A & -\rho_A A & -\rho_A A \\ -\rho_P P & \rho_P(C - P) - \delta_P - \mu_P & -\rho_P P + \delta_N \\ -\rho_N N & -\rho_N N + \delta_P & \rho_N(C - N) - \delta_N - \mu_N \end{bmatrix}.$$

For the non-cancerous fixed point $(A, P, N) = (A_0, 0, 0)$, the characteristic equation $\det(J - \lambda I) = 0$ is

$$(\lambda + \mu_A - \rho_A(1 - 2A_0))((\lambda + \delta_P + \mu_P - \rho_P(1 - A_0))(\lambda + \delta_N + \mu_N - \rho_N(1 - A_0)) - \delta_P \delta_N) = 0.$$

The equilibrium is stable if and only if all solutions have negative real part. The first term gives the solution $\lambda = \rho_A(1 - 2A_0) - \mu_A < \beta_A + \rho_A A_0(1 - A_0) - \mu_A A_0$, which is negative by Eq (C). This leaves the solutions to the quadratic

$$\lambda^2 + \lambda(S_P + S_N) + S_P S_N - \delta_P \delta_N = 0,$$

where $S_P = \delta_P + \mu_P - \rho_P(1 - A_0)$ and $S_N = \delta_N + \mu_N - \rho_N(1 - A_0)$. Thus by the Routh–Hurwitz stability criterion, all solutions will have a negative real part if and only if $S_P + S_N > 0$ and $S_P S_N > \delta_P \delta_N$.

Let $C = C_1$ be the solution of (H), and define $S'_P = \delta_P + \mu_P - \rho_P C_1$ and $S'_N = \delta_N + \mu_N - \rho_N C_1$. We saw above that there exists a physical equilibrium with non-zero cancer exactly when $C_1 < 1 - A_0$ and the P/N ratio given by Eq (G) for $C = C_1$ is positive (note that Eq (G) holds by the definition of C_1). This implies that $S'_P > S_P$, $S'_N > S_N$, $S'_P > 0$ and $S'_N > 0$, and hence either $S_P S_N < S'_P S'_N = \delta_P \delta_N$ or else both S_P and S_N are negative. Thus $(A_0, 0, 0)$ is unstable. Conversely, if $(A_0, 0, 0)$ is stable then $S_P S_N > \delta_P \delta_N = S'_P S'_N$ and $S_P, S_N > 0$. This implies that either $C_1 > 1 - A_0$ or both S'_P and S'_N are negative. Thus there is no real physical solution with $N, P > 0$.

We now suppose that there exists a steady state (A_1, P_1, N_1) with $A_1, P_1, N_1 > 0$, and consider stability at this point. Let $C_1 = 1 - A_1 - P_1 - N_1$ and $R = P_1/N_1 > 0$. By Eqs (C) and (G) we have $\mu_A - \rho_A C_1 = \beta_A/A_1$, $\delta_P + \mu_P - \rho_P C_1 = \delta_N/R$, and $\delta_N + \mu_N - \rho_N C_1 = R\delta_P$, giving

$$\lambda I - J = \begin{bmatrix} \lambda + \frac{\beta_A}{A} + \rho_A A & \rho_A A & \rho_A A \\ \rho_P P & \lambda + \frac{\delta_N}{R} + \rho_P P & -\delta_N + \rho_P P \\ \rho_N N & -\delta_P + \rho_N N & \lambda + R\delta_P + \rho_N N \end{bmatrix}.$$

Noting the cancellation of all terms containing $(\rho_A A)(\rho_P P)$, $(\rho_A A)(\rho_N N)$, or $(\rho_P P)(\rho_N N)$ in $\det(\lambda I - J)$, we obtain the characteristic equation

$$\lambda^3 + a_2\lambda^2 + a_1\lambda + a_0 = 0,$$

where

$$\begin{aligned} a_2 &= \frac{\beta_A}{A} + \rho_A A + \frac{\delta_N}{R} + R\delta_P + \rho_P P + \rho_N N \\ a_1 &= \frac{\beta_A}{A}\left(\frac{\delta_N}{R} + R\delta_P + \rho_P P + \rho_N N\right) + \rho_P P\left(R\delta_P + \delta_P\right) \\ &\quad + \rho_N N\left(\frac{\delta_N}{R} + \delta_N\right) + \rho_A A\left(\frac{\delta_N}{R} + R\delta_P\right) \\ a_0 &= \frac{\beta_A}{A}\left(\rho_P P\left(R\delta_P + \delta_P\right) + \rho_N N\left(\frac{\delta_N}{R} + \delta_N\right)\right). \end{aligned}$$

Since all parameters and state variables are positive, we observe that $a_2, a_1, a_0 > 0$ and $a_2 a_1 > a_0$. Thus by the Routh-Hurwitz criterion all solutions have a negative real part, and the fixed point (A_1, P_1, N_1) is stable.

Thus we have shown that there is either a single, stable fixed point with $P = N = 0$, or else this fixed point is unstable and there is a second, stable fixed point with $P, N > 0$.

References

1. Sharp JA, Browning AP, Mapder T, Burrage K, Simpson MJ. Optimal control of acute myeloid leukaemia. Journal of Theoretical Biology. 2019;470:30–42. doi:10.1016/j.jtbi.2019.03.006.

2. Pontryagin LS, Boltyanskii VG, Gamkrelidze RV, Mischenko EF. The Mathematical Theory of Optimal Processes. New York: Interscience; 1962.
