# Supplementary material for: Optimal control of multiple myeloma assuming drug resistance and off-target effects
Source: PLoS Comput Biol. 2025 Aug 11;21(8):e1012225. doi: 10.1371/journal.pcbi.1012225 (PMC12364355; doi:10.1371/journal.pcbi.1012225)
Supplement: S2 Text — Several sensitivity and other additional analyses. (PDF) [file pcbi.1012225.s002.pdf]

S2 Text - Supplementary Results

James G. Lefevre[1,2*], Brodie A.J. Lawson[2,3], Pamela M. Burrage[2,3], Diane M. Donovan[1,2], Kevin Burrage[2,3,4]

1 School of Mathematics and Physics, The University of Queensland, Brisbane, Australia
2 ARC Centre of Excellence, Plant Success in Nature and Agriculture
3 School of Mathematical Sciences, Queensland University of Technology, Brisbane, Australia
4 Department of Computer Science, University of Oxford, Oxford, UK

* j.lefevre@uq.edu.au

A Optimal controls under variation of drug resistance parameters

The drug resistance mechanism we model consists of four added features: an alternative CD38- cancer state with reduced fitness; switching of cancer cells between the CD38+ and CD38- states; a response to treatment in the form of loss of CD38 expression; and mortality of CD38+ but not CD38- cancer cells in response to the control. Here we consider the sensitivity of the model and the optimal control solutions to the parameters controlling these features. The mortality effect is kept fixed with $\mu_{Pu} = 1$ while we consider variations in the other three features, observing distinct responses in each case.

For the expression switching mechanism, in which cancer cells lose or gain CD38 expression, we retain $\delta_N/\delta_P = 10$ to reflect the typical dominance of the CD38+ state, but consider large coordinated changes in both values (Fig A). With the linear cost, higher rates of switching lead to a prolonged control which is higher in aggregate despite a shorter initial "bang" period at maximum intensity. The temporal pattern of the singular arc also changes: at the highest level of expression switching there is a much stronger reduction over time in the control level. The aggregate cancer level remains relatively constant, although CD38 expression increases. Notably, there is almost no corresponding effect on the quadratic cost optimal control, in contrast to all other parameter variations considered (Figs 4 and 5 in the main text, and Figs B and C). This is likely related to the nature of the change in the linear controls; increasing the switching rates does not greatly increase the length of treatment, and the tapered shapes of the quadratic controls already include a similar period of control at reduced intensity.

The fitness penalty from loss of CD38 expression in cancer cells is represented by a reduction in proliferation and increase in mortality. Plausible variations in the size of this penalty have little effect on outcomes except for a modest reduction in treatment duration at higher fitness penalty (Fig B). However, if the fitness penalty is removed entirely (left side) there is a large increase in cost driven by extended control and persistent CD38- cancer cells. Note that we do not attempt to disentangle the effects of changes in proliferation and mortality.

Finally, the most complex response is elicited from variation in the rate of drug-induced loss of CD38 expression (Fig C). Optimal control solutions appear to show competing effects from this loss of CD38 expression: the drug control induces a CD38- subpopulation that persists under treatment, imposing a health burden and requiring

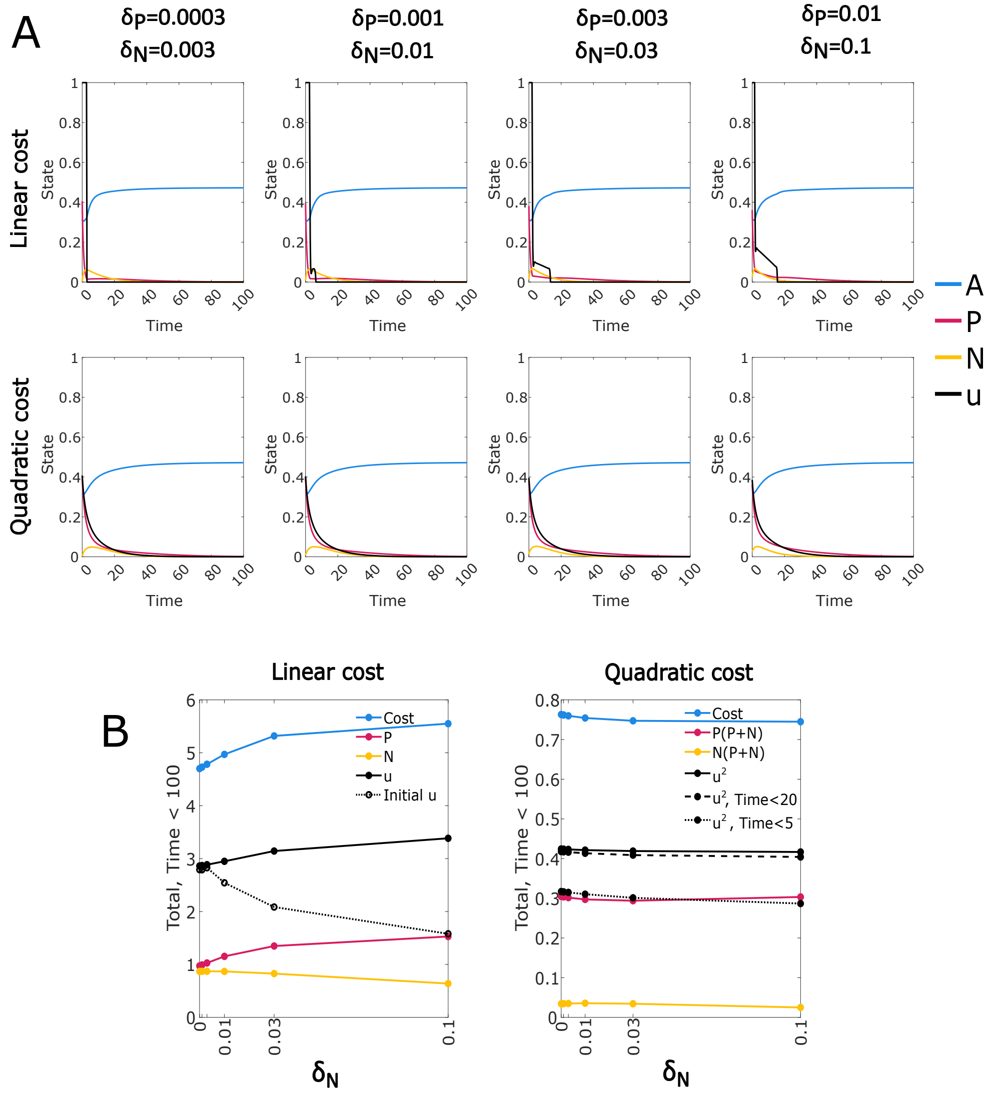

Fig A. Effect of expression switching of CD38 expression on optimal treatment: optimal control solutions for range of δ_P and δ_N values (default values $\delta_P = 0.003, \delta_N = 0.03$). For the linear cost function a higher switching rate corresponds to more prolonged treatment (extended singular control period). Only minor changes are seen for the quadratic cost function controls. A: Linear and quadratic cost optimal control solutions, selected values of δ_P and δ_N. B: Total costs and components up to Time=100; note $\delta_P = \delta_N/10$. Each value plotted is the integral of the specified function over the interval Time $\in [0, T]$, where $T = 100$ unless otherwise specified.

more prolonged treatment (particularly for the bang-bang control). However, the lower fitness of this subpopulation results in a relatively stable or reduced quantity of control required in aggregate.

B Sensitivity analysis for expression switching ratio

The model was designed to ensure a predominance of P over N in the absence of a forcing mechanism such as the drug control. We assumed that this predominance resulted from a combination of lower fitness in N and a much higher propensity to gain than to lose expression ($\delta_N/\delta_P = 10$). While we considered the effect of varying the fitness differential (Fig B), the δ_N/δ_P ratio was conserved even when we considered the effect of changing the general rate of expression switching (Figs 6,7,8, A). Since this ratio is purely an assumption, we briefly test the effect of reducing the ratio. We fix each of the default values $\delta_N = 0.03$, $\delta_P = 0.003$ in turn, and reduce the ratio to 3 and then 1 (Fig D). Comparing with Fig B, we see that when the ratio is 3 there is minimal difference from the corresponding case with ratio 10 and the same value of δ_N, suggesting that there is limited sensitivity to the specific value of δ_N/δ_P ratio provided that it is significantly greater than 1. More substantial differences are apparent when $\delta_N = \delta_P$, although we still produce bang-singular controls for the linear cost function, and see no major differences in control form. The initial value of N in these cases remains relatively low, implying that the fitness difference is sufficient to maintain the required predominance of P in the absence of the control. Therefore, a model with $\delta_N = \delta_P$ is plausible, although we do not investigate further in this work.

C Cost function sensitivity analysis

The scope of the primary analysis is restricted to two cost functions, linear and quadratic, each with equal weighting of the drug and cancer terms. Parameters were chosen so that the cost contributions of the two terms should be comparable in both cases, and there is an implicit assumption that there is only a moderate sensitivity to the weighting of the terms, such that any general conclusions can be expected to hold under reasonable changes to the weighting. By considering both linear and quadratic cost functions we seek to identify robust trends alongside patterns which are specific to each case. Although the true cost structure is unknown, it can reasonably be supposed that it could be more accurately approximated by some generalised cost function containing both linear and quadratic terms. Thus our approach also implicitly assumes that optimal control solutions for such a generalised cost function would be broadly intermediate between the linear and quadratic cost function cases, rather than exhibiting a distinct form. In this section we test these assumptions for the default model parameters.

To test sensitivity to the weighting of drug and cancer costs, we replace the cost functions (7) and (8) in the main text with (A) and (B) respectively, using values of $W_C = 0.5, 1, 2$.

$$\textbf{Weighted linear cost:} \quad \mathcal{L} = u + W_C(P+N), \text{ where } 0 \leq u \leq 1. \tag{A}$$
$$\textbf{Weighted quadratic cost:} \quad \mathcal{L} = u^2 + W_C(P+N)^2. \tag{B}$$

The costate equations in Section B in S1 Text are modified by replacing the $-2(P+N)$ term in the equations for $\frac{d\lambda_2}{dt}$ and $\frac{d\lambda_3}{dt}$ with $-2W_C(P+N)$. Similarly, the costate equations in Section C in S1 Text are modified by replacing the corresponding -1 terms with $-W_C$. The results are shown in Fig E. We see only moderate changes in the optimal controls in the expected direction, with increased weighting of cancer cost associated with moderately increased duration and intensity of treatment, and corresponding reduction in cancer levels. We also confirm that the cost contributions from the drug and cancer terms are of comparable magnitude.

To test the behaviour of the optimal control under a generalised second order cost function, we consider the cost function (C). We use the computational strategy described in Section B in S1 Text.

$$\textbf{Generalised cost function:} \qquad \mathcal{L} = u + P + N + \kappa\left(u^2 + (P+N)^2\right). \qquad \text{(C)}$$

The costate equations are modified by replacing the $-2(P+N)$ term in the equations for $\frac{d\lambda_2}{dt}$ and $\frac{d\lambda_3}{dt}$ by $-1-2\kappa(P+N)$. Similarly, the u^* equation is modified with the addition of the term -1 in the numerator, and a denominator of 2κ instead of 2, and we constrain the updated control function to the interval $[0,1]$. In Fig F we show the optimal control solutions for default model parameters and $\kappa = 1, 0.1$, alongside the original cost functions. The case $\kappa = 1$ represents a simple sum of the linear and quadratic cost functions, and gives an intermediate control form consistent with our assumption. The case $\kappa = 0.1$ represents a predominantly linear cost function, and we obtain an optimal control that approximates the form of the bang-singular solution for the linear cost. This result supports broad continuity in the solution space despite the modified computation method required for the linear cost, and therefore provides important validation of the intermediate level controls found in the linear cost case, and the method used to generate them.

D Extended and infinite horizon validation

In this section we check the assumption that the optimal controls shown (either here or in the main text) are not influenced by finite time window effects, and can be reasonably extrapolated to an indefinite treatment regime. We take two approaches. Firstly, we compare the final value of each computed optimal control to the infinite horizon optimum. We also recalculated selected optimal controls on a double length ($t \in [0, 400]$) time window. This is compared to the standard control calculated on $t \in [0, 200]$, over the evaluation interval $[0, 100]$. See Section D in S1 Text for methodology.

The infinite horizon time-averaged cost optimal control is the level of the control u such that this value and the corresponding stable equilibrium of the system minimises the function $\mathcal{L}$ given by Eq (8) (quadratic cost) or Eq (7) (linear cost) in the main text. In all cases with $\alpha > 0$ the computed optimal control shows the control and cancer level at or approaching zero by the end of the time window, implying no ongoing costs. In each case this was confirmed by an infinite horizon minimum cost solution $u = N = P$, confirming that no delayed recurrence would occur under our model. In the remaining cases, in which $\alpha = 0$, the infinite horizon optimum is shown in Table A alongside the computed optimal control and corresponding system values at $t = 100$. Note that all these cases, referenced below by figure number, were presented in the main text.

In 6 of the 14 cases (Fig 4A, top and bottom left, Fig 6, bottom left, Fig 7, bottom right, Fig 8, panels 2 and 4) the computed optimal control has reached or is approaching the infinite horizon optimum. In the three cases with an ongoing cyclic form (Fig 6, top left, Fig 7, bottom left and centre), the infinite horizon optimums appear to be consistent with the cyclic solutions, although they cannot validate them directly. This leaves two special cases. Firstly, the top row of Fig 7 has the condition $\delta_P = \delta_N = 0$. This acts to disconnect the P and N populations, except for a one-way transfer from P to N if u and δ_{Pu} are both positive. With $\alpha = 0$, the P population can eventually recover from levels arbitrarily close to zero; this is biologically implausible, as noted in the discussion of Fig 4 in the Results. A second edge case is seen in Fig 8, panels 1 and 3. With $\delta_{Pu} = 0$ and very low levels of expression switching, the N population can increase only very slowly from its initial low level. As a result, in this case the apparent stability at $t = 100$ is illusory, and convergence to the true steady

			Control solution			
Figure	cost	case	u	A	P	N
Fig 4A, top left	linear	$t = 100$	0.1198	0.4148	0.0338	0.0649
		∞	0.1368	0.4337	0.0154	0.0364
Fig 4A, bottom left	quadratic	$t = 100$	0.1153	0.4131	0.0373	0.0664
		∞	0.1183	0.4253	0.0271	0.0480
Fig 6, top left	linear	$t = 100$	0.0000	0.3658	0.0609	0.1946
		∞	0.0641	0.3360	0.0488	0.2151
Fig 6, bottom left	linear	$t = 100$	0.0339	0.3450	0.0178	0.2535
		∞	0.0347	0.3470	0.0170	0.2478
Fig 7, top left	linear	$t = 100$	0.0000	0.4727	0.0000	0.0000
		∞	0.0640	0.3360	0.2640	0.0000
Fig 7, top centre	linear	$t = 100$	0.0000	0.3791	0.0002	0.2162
		∞	0.0533	0.3421	0.0000	0.2579
Fig 7, top right	linear	$t = 100$	0.0000	0.3701	0.0001	0.2392
		∞	0.0213	0.3617	0.0000	0.2383
Fig 7, bottom left	linear	$t = 100$	0.0000	0.4439	0.0195	0.0386
		∞	0.0705	0.3406	0.1453	0.0971
Fig 7, bottom centre	linear	$t = 100$	0.0000	0.4266	0.0388	0.0219
		∞	0.1284	0.3889	0.0248	0.0165
Fig 7, bottom right	linear	$t = 100$	0.1290	0.4040	0.0007	0.0001
		∞	0.1206	0.4093	0.0000	0.0000
Fig 8, panel 1	linear	$t = 100$	0.0942	0.4582	0.0101	0.0208
		∞	0.0716	0.3872	0.0998	0.0951
Fig 8, panel 2	linear	$t = 100$	0.0719	0.3887	0.0257	0.1663
		∞	0.0698	0.3790	0.0298	0.1864
Fig 8, panel 3	linear	$t = 100$	0.0955	0.4449	0.0127	0.0251
		∞	0.0713	0.3768	0.1099	0.0956
Fig 8, panel 4	linear	$t = 100$	0.0703	0.3774	0.0297	0.1754
		∞	0.0682	0.3689	0.0340	0.1934

Table A. Optimal control values at $t = 100$ compared to infinite horizon time-averaged optimum, in all cases where this is non-zero. In general, our calculated optimal controls converge over time towards the long term treatment level that minimizes time-averaged cost. However, this table highlights two cases where this fails: when $\delta_P = \delta_N = 0$, removing a core part of the model (Fig 7, top row); and controls with an indefinite cyclic form (Fig 6 top left, Fig 7 bottom left and center). We also note that in some cases convergence is incomplete, and these directly computed infinite horizon solutions may provide a useful check. All figures refer to the main text.

state solution occurs over a far longer time period. We note that both of these special cases occur when a core part of the drug evasion mechanism is removed.

In Fig G we compare selected optimal control cases calculated on the standard $t \in [0, 200]$ time interval with the result for a double length interval $t \in [0, 400]$. The two panels on the right were selected due to incomplete convergence to the $u = N = P$ steady state over the $t \in [0, 200]$ window, likely a result of the weakened immune response ($\alpha = 0.01$). The extended time window produced much improved convergence and significant change in the optimal control, although the general form was unchanged. In these cases, the extended time window control was used in the primary figure. It is clear that the optimal control using our approach can only be trusted to the degree that convergence to the infinite horizon optimum has been achieved. The other case to exhibit a substantial difference on the extended window was the ongoing cyclic solution.

The end of time window effect will suppress the control level close to the final time, which will tend to influence the timing of the treatment cycle. This is consistent with the difference observed, and a similar effect can be expected with other cyclic control cases. The general form of the control is unchanged, but if the exact optimal control is required in these cases alternative methods may be required.

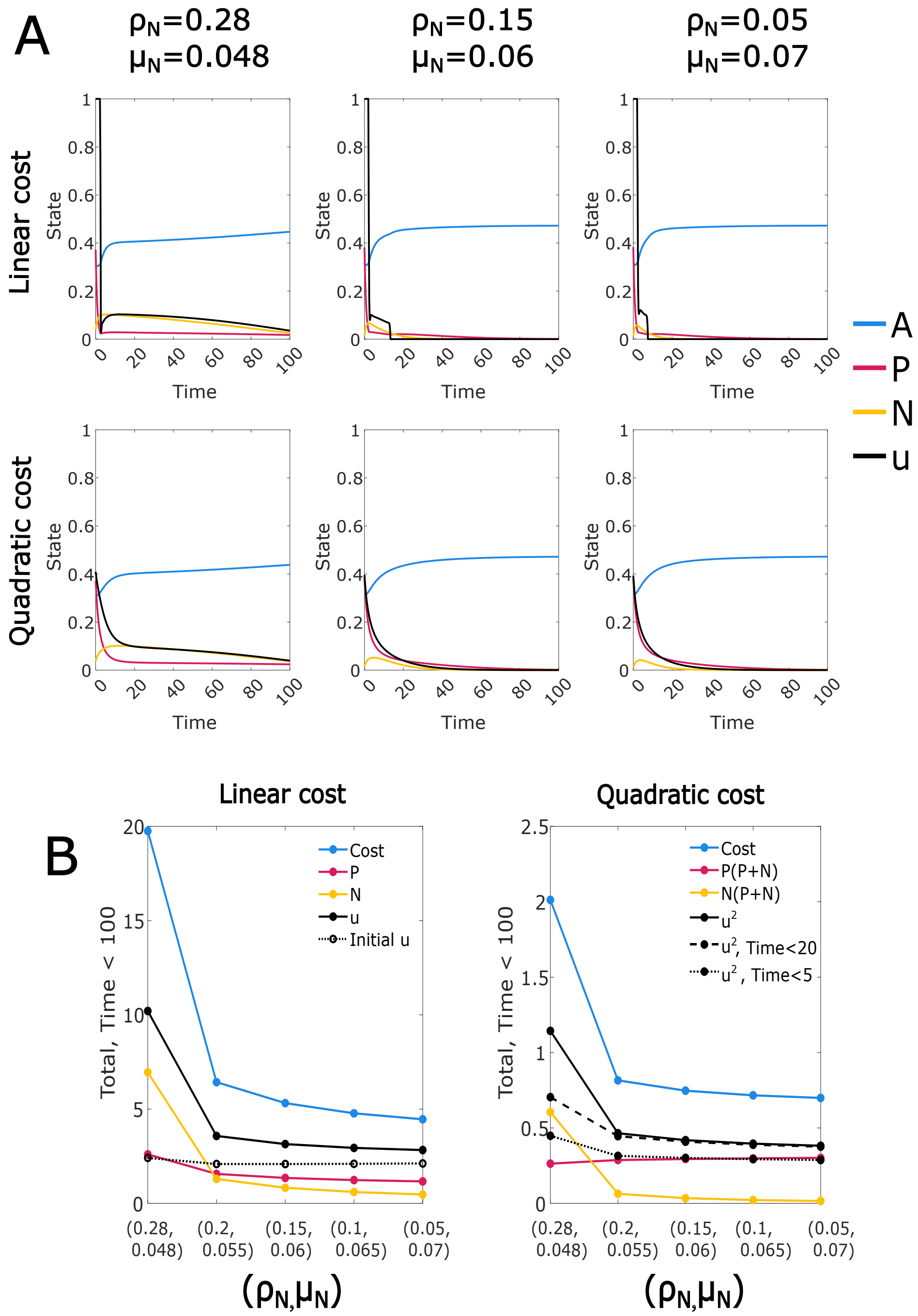

Fig B. Effect of CD38- myeloma (N) fitness on optimal treatment: optimal control solutions for a range of ρ_N and μ_N values. The fitness of CD38- cancer cells decreases across the subplots (left to right), with the leftmost value representing fitness equal to the CD38+ cells (original values for CD38- cells $\rho_N = 0.15$ and $\mu_N = 0.06$). For both cost functions, increased N fitness corresponds to more prolonged treatment and higher overall costs, especially as fitness reaches the same level as P. A: Linear and quadratic cost optimal control solutions, selected values of ρ_N and μ_N. B: Total costs and components up to Time=100. Each value plotted is the integral of the specified function over the interval Time $\in [0, T]$, where $T = 100$ unless otherwise specified.

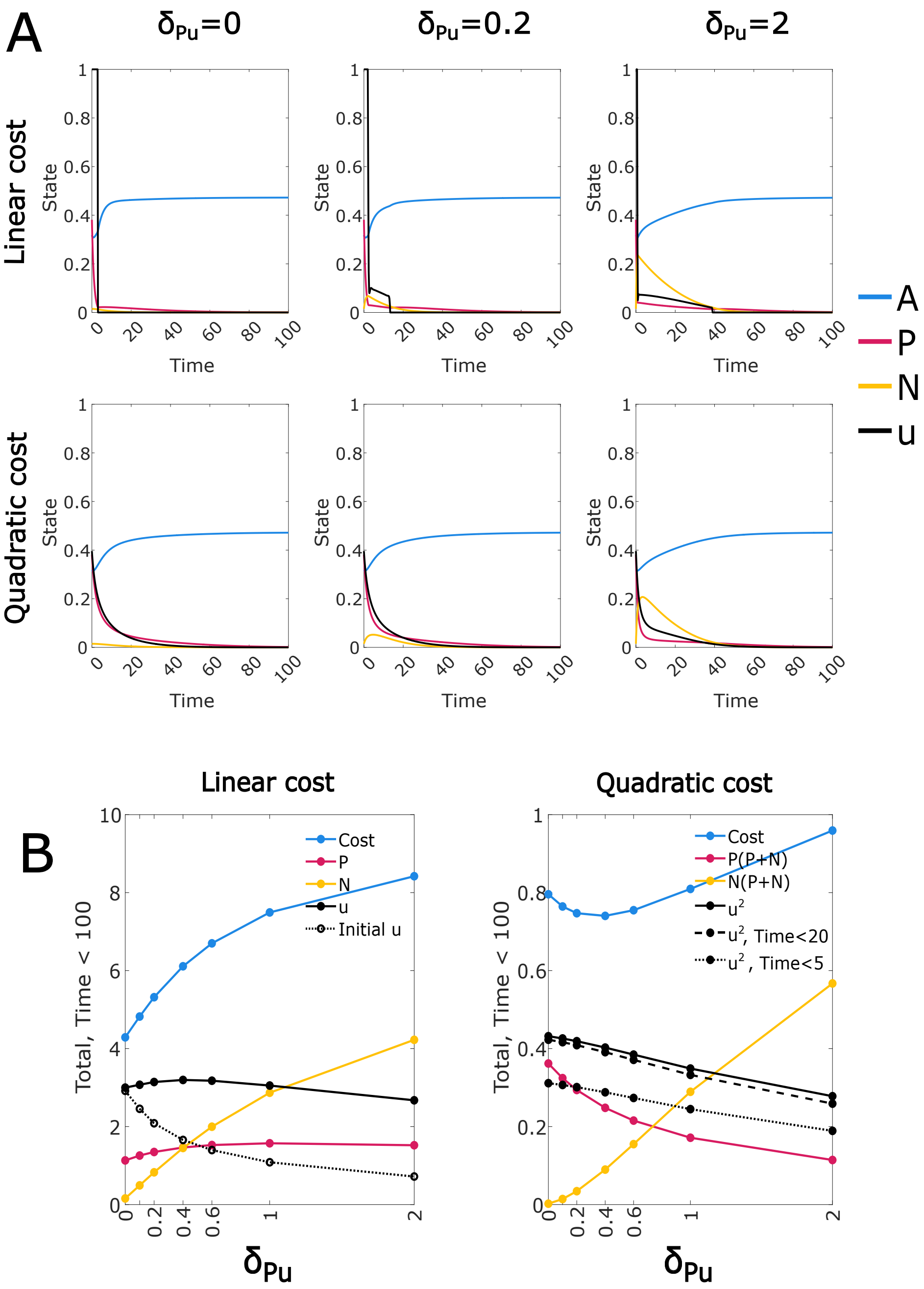

Fig C. Effect of drug-induced loss of CD38 expression on optimal treatment: optimal control solutions for range of δ_{Pu} values (original value $\delta_{Pu} = 0.2$). Higher values mostly correspond to reduced drug treatment but higher overall cost, with the exception that minimum cost for the quadratic function and maximum total control with the linear cost function are reached at intermediate values. The higher rate of drug-induced loss of expression also corresponds to more prolonged treatment for the linear but not the quadratic cost function. A: Linear and quadratic cost optimal control solutions, selected values of δ_{Pu}. B: Total costs and components up to Time=100. Each value plotted is the integral of the specified function over the interval Time $\in [0, T]$, where $T = 100$ unless otherwise specified.

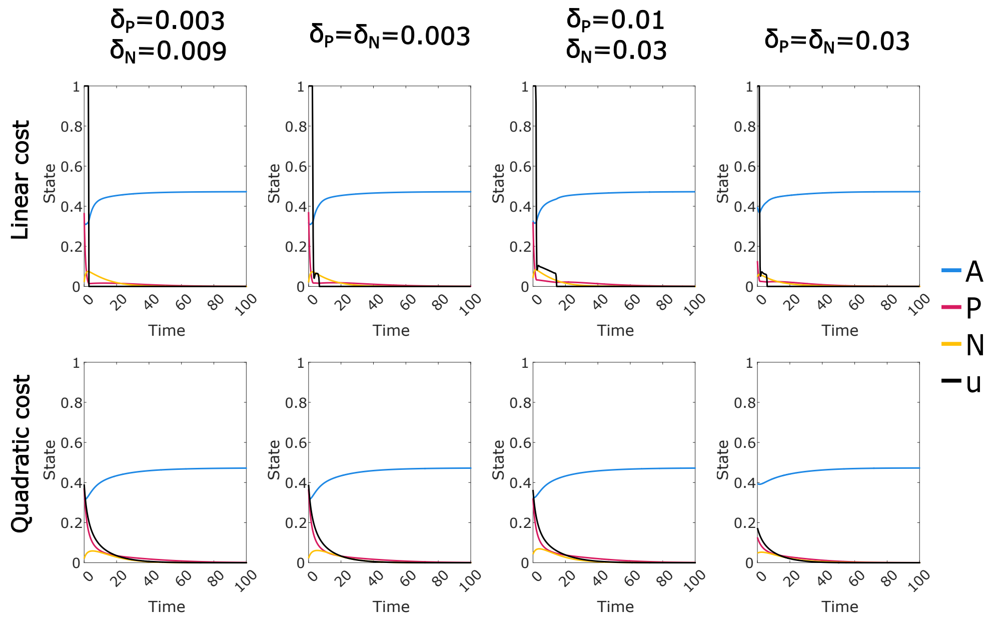

Fig D. We consider the effect of reducing the ratio δ_N/δ_P from the default value of 10 to 3 or 1.

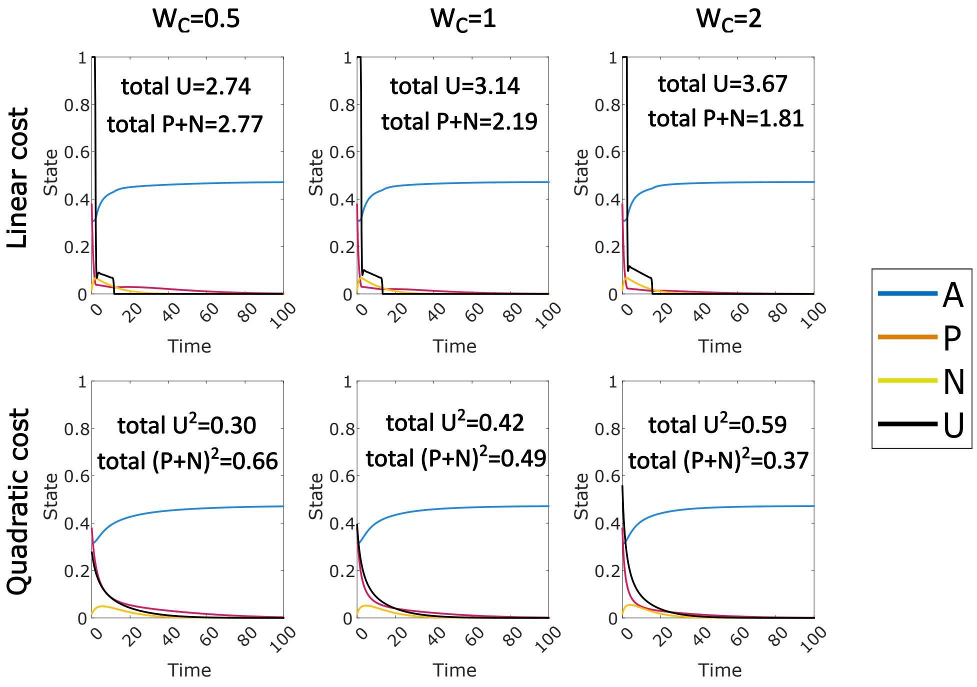

Fig E. Effect of changing relative weightings of cost function components by factor of 2 in each direction (original value $W_C = 1$). Control solutions show limited sensitivity to the cost weightings, changing moderately and in the expected direction.

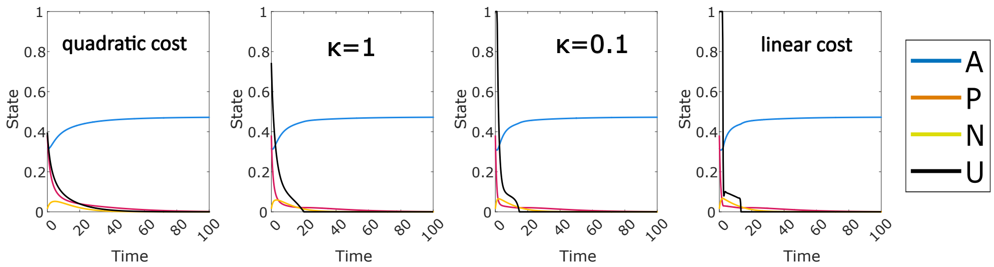

Fig F. Optimal controls for generalised super-linear cost function (C) and default model parameters. Combining linear and quadratic cost terms yields an intermediate control form.

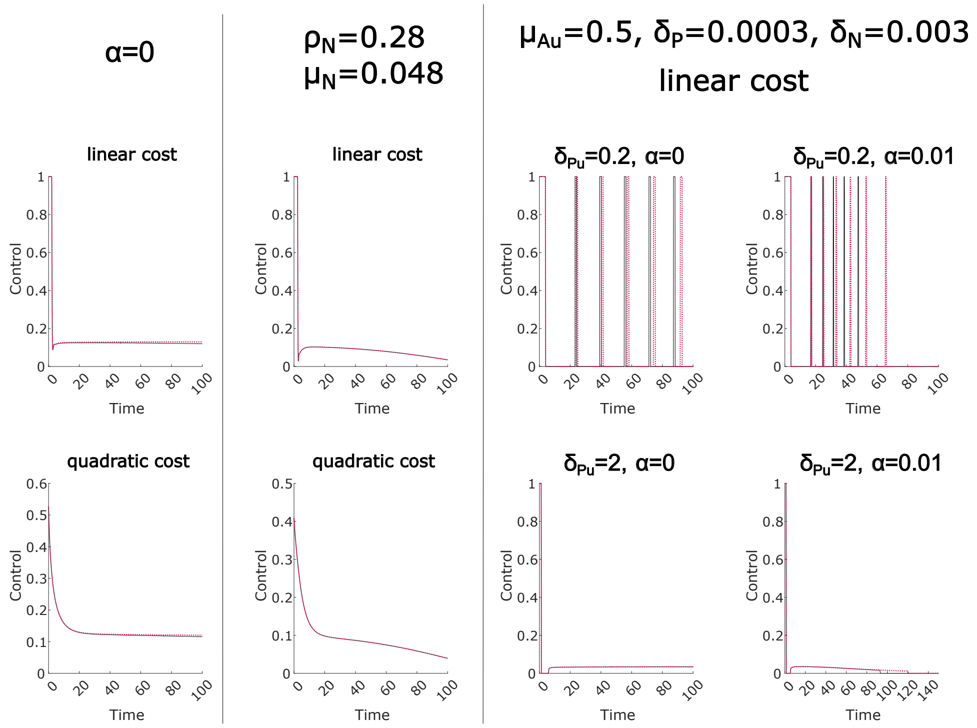

Fig G. Comparison of optimal controls calculated on standard interval ($t \in [0, 200]$, solid black line) and double length interval ($t \in [0, 400]$, dotted red line). The controls are compared on the interval $t \in [0, 100]$, or in one case $t \in [0, 140]$. The comparison was performed for selected cases: Fig 4 left top and bottom (shown in first column); Fig B left top and bottom (second column); and Fig 6 left and centre panels (third and fourth columns). We see minimal evidence of finite time window artefacts except in the case of the ongoing cyclic controls and in the two cases with $\alpha = 0.01$ where convergence to the infinite horizon steady state was incomplete at $t = 200$.
