# Supplementary material for: Optimal control of multiple myeloma assuming drug resistance and off-target effects
Source: PLoS Comput Biol. 2025 Aug 11;21(8):e1012225. doi: 10.1371/journal.pcbi.1012225 (PMC12364355; doi:10.1371/journal.pcbi.1012225)
Supplement: Attachment — Submitted filename: Optimal Control Plos Comp Bio Review - to Authors.pdf [file pcbi.1012225.s003.pdf]

Optimal control of Multiple Myeloma assuming drug evasion and off-target effects

Summary

The authors proposed to use optimal control techniques to derive treatment schedules, within the disease context of multiple myeloma. This was conducted on an ODE model of a partially drug resistant tumour, building on previous work by Khalili et al. and Sharp et al. (who are cited in the manuscript). Novel to this paper, the authors also allowed for both drug evasion (via reversible CD38 expression) and off-target killing of healthy cells.

The inclusion of these additional phenomena is well motivated within the study. It is worth noting that the study is purely theoretical (neither the ODE model nor the derived treatment regimens are parameterised by nor compared to experimental/clinical data), however the authors do well to convey those limitations. The authors also provide code for their model, although I have detailed difficulties in running this below.

These additional drug dynamics motivate quantitative (i.e. extended treatment times in Sections 2.2 & 2.3) and qualitative changes (Sections 2.5 & 2.6) to the optimal treatment approach. The latter are particularly exciting, demonstrating that optimal binary dosing strategies may consist of either single or multiple treatment periods depending on the parameter regime. Within the context of this class of models, this appears to be the first demonstration of optimal cyclic controls.

However, the authors employ an unconventional form of optimal control, that I believe is mis-categorised in the paper as ‘bang-bang’ control; I provide more details of prior approaches to cancer therapeutic scheduling using conventional ‘bang-bang’ control below. I recommend that the authors reframe the narrative of this paper to focus on the impact of different objective cost functions (within a continuous control framework). This would build upon established literature in the field, and I believe would also present their current results through a stronger narrative which could be of wider interest.

Significant Revisions

I have split the body of this review into more significant, and minor, revisions. While I appreciate the review is lengthy, I hope that this will make the content more digestible, and I firmly believe it will strengthen this interesting paper. Additionally, I have endeavoured to signify minor revisions that may require (small) additional analysis or visualisation changes by '**' and '*' respectively, with other revisions relating purely to the text itself. I hope that this will support the authors when revising this manuscript.

In general, this paper would benefit from further context of previous work applying optimal control to immune/targeted therapies, and additional clarity on the novel contributions of this paper relative to previous work in the field. In particular, I would like to draw the authors attention to recent work by Khailov et al. (https://doi.org/10.3390/g11040053) that builds on the cited work by Grigorenko et al. and applies optimal control to a Leukaemia treatment model. They identify different regimes in which continuous or intermittent therapies (under bang-bang control) are optimal, akin to the results in the final results of the submitted manuscript (though these results only consider single breaks in treatment, rather than the cycling behaviour explored by the authors). More recently, Ledzewicz et al. (https://doi.org/10.3934/dcdsb.2023141) have integrated a similar approach with the relative weighting approach I mention below. The authors may find it beneficial to compare with these approaches to bang bang control, which avoids the problem of intermediary doses that the authors experienced (though is not without its own drawbacks).

In this vein, my primary concern is that the formulation of 'bang-bang' control in this paper does not seem to match with existing literature, such as traditional formulations (https://doi.org/10.1016/0022-247X(63)90081-7, https://www.jstor.org/stable/43634288), and prior literature applying conventional bang-bang control to chemotherapeutic scheduling (https://doi.org/10.1023/A:1016027113579). I believe this is the cause for the intermediary control values observed in Sections 2.4 onwards.

I therefore have to disagree with the final paragraph of Section 2.4 - to my understanding this behaviour is not typically present in bang-bang control because bang-bang control is traditionally formulated to prevent this behaviour (the control is assigned according to the sign of the switching function - see refs above). Because these results could not be replicated by a conventional method of bang-bang control, it seems to me that they are necessarily an artefact of the method used here. Indeed, the conventional formulation of 'bang-bang' control, wherein the control is determined by a switching function, has also been adopted by Sharp et al. (whose work is cited in this paper as the basis for the authors' approach). This work appears to avoid the issue of non-binary controls above - while I see this may be more challenging to formulate for the cycling solutions in Section 2.6, it would certainly help the convergence issues in previous sections.

I should emphasise that I do not think it is necessary for the authors to reformulate this control method, though the authors may pursue this if they wish. However, it may be more accurate to describe this as a different form of continuous control, with a linear control function designed to favour (but not limited to) extremal values. I believe this would offer a compelling overall narrative, comparing the impact of different cost functions instead of

different control methods to show that this qualitatively changes the forms of the derived control. For some context on prior work addressing this question, I recommend https://doi.org/10.1007/s10957-020-01754-2; https://doi.org/10.1016/j.mbs.2010.11.007. In particular, the authors may wish to comment on the generalisability of this approach to other cancer sites or therapeutic agents in the discussion. It might be interesting to note that some drugs are conventionally approved at multiple dosing levels (or even across a dosing spectrum); the authors can therefore argue that the cost function could be modified based on the drug context, with quadratic functional forms being more appropriate to drugs with multiple dosing levels than linear forms.

This would also feed into the authors' wider narrative on the functional forms of the cost function. I think this is a very interesting area that is not done justice in the current manuscript; the cost functions are introduced with minimal explanation for their functional form in Section 1.3. The functional forms are commented on further in Section 2.2, explaining that the linear form is required for the algorithm to favour binary dosing, but this doesn't explain the quadratic form of the drug cost, given it is criticised in L218, and later in L374. While I have no qualms with the use of a quadratic cost function given its importance within the optimal control literature, the authors should be explicit whether this is motivated by any real-world application or simply a mathematical requirement for the optimal control framework. This will help reconcile the use of this approach with the authors' own recognition that this cost function differs significantly from reality, and help persuade the reader that a control solution (treatment schedule) that minimises the square of the daily drug cost (instead of the cumulative drug cost) has real-world relevance.

Additional comparison of the cost functions used would also be helpful - both linear and quadratic cost functions are used in this study, but the circumstances under which each method should be preferred are not clear. Perhaps linear cost functions are more appropriate when binary dosing treatment schedules are desired (due to clinical restrictions?) The authors could also argue that the linear dose function has helped them identify mechanisms within the ODE model that affect the optimal control, whereas this would not have been possible with the quadratic function. This could also extend the applicability of this work to other cancers/biological systems outside of multiple myeloma, if the linear cost function is promoted as a method to identify which mechanisms within a complex model affect the model's optimal control.

In further discussion of the cost function, the authors recognise that the correct weighting between the health cost of the cancer and the financial cost of the drug is not obvious. However they then sum these two costs with no additional scaling, in effect assuming a very specific weighting between these two costs. While I recognise that this would be a major change, modifying the cost functions to include a tunable parameter that scales the relative weighting of these two costs (i.e. by replacing the drug cost 'u' with ' beta u' for some unknown beta) would generate significantly more generalisable solutions - see Sharp et al. for an example of this approach. The authors could then verify that their qualitative conclusions are not dependent on the value of beta that they have implicitly assumed (either through a systematic evaluation of the system when beta is varied, or simply by selecting a discrete number of values of beta to test). At the very least, I think that the authors need to be explicit that an implicit balance between these two costs has been chosen here, though I do feel that that reduces the strength of the paper's conclusions somewhat.

In addition to these comments on the cost function, I have a few other significant suggestions for the paper, particularly in mind of the typical style and format of papers in PLOS Computational Biology, and the connection to biological systems.

I found Section 2.4 very confused - in comparison to Sections 2.5 & 2.6 which I thought were excellently written, I wasn't clear on the salient points from this section. I have included some suggestions in the line-by-line comments below on how to address this and simplify that section, but I wondered whether this section might be better placed in the Supplementary Information? This might also mitigate a confusing section of the discussion (L347-350), where the authors mention the mixed results from Section 2.4 but struggle to motivate or explain them further. I have provided further suggestions on this section (including the figures) in minor points below.

I also suggest that the authors review their figure captions; I think it would be helpful for the figure captions to highlight the important trends that a reader should be looking for in these figures in addition to simply labelling the figure. For example the caption for Figure 4 simply states these are optimal control solutions, but could also draw the reader's attention to the reduced drug duration under both control methods as immunity is strengthened (left to right). It would also be helpful to remind the reader what each parameter refers to in these captions (throughout the paper), particularly those that are crucial to understanding of the figure (such as those varied between subplots), so the reader does not need to refer back to the original table - authors could label figures/axes/subpanels with the variable names (with the symbols in brackets) and indicate how model behaviour changes between subpanels descriptively (i.e. 'Increasing Immunity Strength' on an arrow going left -> right in Figure 2a).

Furthermore, while I felt that the limitations that the model assumptions placed on the applicability of this work were well described, I detail below some of the methodological decisions that also seem to limit the applicability of this paper to real-world systems, and would benefit from further explanation/justification. In particular, in Section 2.6, it seems drastic to remove the off-target effect completely (L331), especially as the authors argue previously that this is an important biological effect to include. Can the authors show that the cyclic solution vanishes when the off-target effect is restored to reasonable levels (such as $\mu_{Au} = 0.1$, instead of setting μ_{Au} to zero? Alternatively if the cyclic solution only re-emerges when $\mu_{Au} = 0$ then this should be stated.

Finally, the authors have also provided code with their manuscript. I have been able to verify that the simulation examples script runs without error, and replicated results from Figures 2. However I encountered issues with the optimal_control_example file, and was only able to replicate Figure 3 as well as some panels from Figures 9. Matlab repeatedly threw errors when saving a png version of the figure generated (such as in L69). This is attributed to an invalid figure handle (I suspect due to a terminated simulation being handled internally), and I recommend the authors investigate this further. I was using 64-bit Matlab R2023a (9.14.0.2206163).

Minor Revisions

As mentioned above, revisions that may require (small) additional analysis or visualisation changes are denoted by ‘**’ and ‘*’ respectively, with other revisions relating purely to the text itself.

L13 - The phrase ‘we identify a general increase in treatment duration and costs’ is unclear, and would benefit from further context - what change has resulted in this increase? Or does this statement refer to an observation that optimal strategies typically have longer treatment duration and higher costs (in which case the text could be updated to reflect this)?

L45 - From a clinical perspective, it would be worth clarifying whether mono-therapeutic Daratumumab is typically applied as a first line therapy, or later in treatment (to tumours that have already been exposed to multiple drugs and potentially metastasised). Similarly, it would be helpful to explain whether Daratumumab is only used continuously in the clinic, or whether intermittent treatment regimes have already been explored clinically?

L46 - The authors switch between the terms ‘tolerance’, ‘resistance’ and ‘avoidance’ somewhat interchangeably - to be more in line with cancer literature I would advise using the term ‘resistance’ (e.g. see https://doi.org/10.1038/nrmicro.2016.34).

L49 - Terminology such as ‘occur passively’ is used in a slightly vague/confusing manner. The authors could do more to differentiate between Darwinian evolution (changes in species abundance through means of natural selection), and Lamarckian evolution (individuals actively adapting to the environment, through mechanisms such as drug-induced switching, and passing the changes on to off-spring).

L53 - It is not clear which off-target effects are being referred to here.

L54 (and elsewhere in introduction) - If the authors are referring specifically to the financial cost of treatment here, then it might be helpful to clarify this (or otherwise to explain which costs they consider within scope for this paper).

L62 - The topic of maintenance treatment has not really been addressed previously in this paper, and is not discussed again in the paper. If the authors wish to retain this reference, more explanation of this treatment context would be helpful.

L64 - This introduction explains optimal control very well, but is likely too general for the intended audience, and could be greatly shortened.

L100 - I wish to commend the authors on the labelling of equation (3), which aids the clarity of the text significantly.

L128 - The authors should clarify what data was used to calibrate the model described here, as the cited papers do not give detail on this.

**L135 - Could the authors indicate the timescale of this divergence? Given this system is not necessarily assumed to reach steady state, transitory effects may well be important.

L141 - The authors assume that ‘fitness is significantly lower in N’, however it is not clear how this manifests in the model - additional clarity is needed on what parameter values are affected by this assumption. I assume this is the reasoning for $\rho_{P} > \rho_{N}$? And is there any evidence for this fitness cost?

L143 - This is an excellent point, and indeed a key differentiation between the authors’ work and Sharp et al. ([27]) - I think that further explanation of why this interaction with population dynamics is beneficial would strengthen this paper.

L145 - The state variable A has not been defined at this point in the text (and I believe is only ever defined implicitly in Table 1) - a more explicit definition in the model description would be helpful.

L150 - The inclusion of a complete table of parameter values is much appreciated! It would be helpful to indicate the providence of these values, so the reader can assess which values were taken from the Sharp model and which were defined by the authors. Given some values have been modified from the Sharp model, perhaps that process could be explained in Supplementary Information/Methods - for example it is not clear how the drug killing parameter values were updated, as drug is assumed to only affect stem cells in the Sharp model, while the authors do not model stem cells explicitly.
Additionally, I could not find numerical values for the initial population sizes used - perhaps they could also be included in this table?
Finally, I found that description “Rate of loss in CB38 expression in P” slightly confusing (as this implied to me that this is an internal transition within the P state) - perhaps the authors could consider rephrasing to “Rate of loss of CB38 expression from P (to N)” or something akin to this?

L172 - Additional justification is required for the removal of the final state cost, and particularly the assertion that this results in more tractable computations - can the authors provide a reference for this statement?

*L190 - Particularly in Figs 2.b and 2.f, it does not appear that the A and P populations have reached steady state - while it seems plausible that they might in the near future it is also not implausible that A might decrease to extinction if simulated for sufficiently long. If the authors wish to state that these populations have reached a balance, I think it would be necessary to show a longer timescale on these plots, as currently they don’t seem to agree with the text.

*L196 - Similarly, Fig 2.h doesn't provide any visual evidence for the claimed reduction in drug efficacy compared to Fig 2.d. While the N population here is non-zero, there is no visually perceptible impact on the A/P populations, nor the timescale over which the cancer (P+N) is eliminated.

L199 - This begs the question, why include the off-target effect in the first place? I think the authors address this very well in Section 2.5+, but should signpost that section here, so that the reader can easily see why this effect was included in your model.

L224 - I am not clear why this provides support for extended treatment? It seems more intuitive to me that this would provide support for applying continuous control over bang-bang control, as a cost function designed to favour binary dosing (u = 0 or 1) has instead selected an intermediary value.

L232 - It isn't clear what the authors mean by burden in this context - the tumour burden is typically used to refer to the number of cancer cells/amount of cancer cells in the body. In that sense, the burden of N cells is decreasing here - but perhaps the "role" of N cells is significant as they can persist under treatment, resulting in a tumour that cannot be eliminated. This would then agree with the excellent later explanation given around L240.

L248 - I am somewhat confused by this point - it seems that the authors are referring to a common phenomenon in ODE models wherein a cell population may decay exponentially, but cannot be fully eliminated due to the continuous nature of the model. While this flaw of the Null-N model is not catastrophic to its biological realism in my view, I recognise that it does pose issues for optimal control. However, to argue that the author's model is superior would require them to show that their model does not exhibit this behaviour - while this may indeed be the case, I can't see it in this section. Perhaps the authors could make a more explicit comparison between the problematic behaviour they see in the two models, and provide a more detailed explanation of how this behaviour practically affects the biological realism of the models.

*L260 - I did not find the b panels very helpful in Figures 5-7. I would recommend that the authors reconsider whether these are essential to the main narrative, or could perhaps be removed/moved to the supplementary information? If the authors do wish to retain these subpanels in the main text, I think that additional description is necessary to explain the novel results/insights presented in these subpanels, that could not be inferred from the other subpanels.
This could also help towards condensing Figures 5-7, as they currently comprise of 22 subpanels which seems gratuitous for their role in the paper, and makes them harder to interpret. For example, from the 8 panels in Fig5a, the only trend I can see (and is commented on in the text) is that the transition from u=1 to u=0 is extended as the switching rate P <-> N increases, which could be conveyed in a single panel depicting these four curves for u. This would help isolate and emphasise this important result, and also provide space for a second subpanel displaying the trend in aggregate control, which the authors mention in the text but do not show.

**L265 - Figure 5 displays some counterintuitive results, which I think warrant further explanation. We see an extended time at intermediate dosing levels, which the authors claim results in a higher aggregate drug level despite the shorter initial duration of maximal drug (aggregate drug levels are not shown though this might be helpful). Yet the aggregate control is not (visually at least) raised under continuous control - I can appreciate that the two control methods will distribute drug in different ways, but why does one method need more drug to cope with increased P <-> N switching whereas both the magnitude and timing of drug is completely unaffected under the other control? We also see this trend in Bang-bang control in Figs 6/7, but there the drug profile in Continuous control is also modified accordingly.

**L273 - From a point of interest, has any attempt been made to disentangle these effects? Presumably it would be fairly quick to rerun this analysis while holding either proliferation or mortality constant - the addition of even a short comment saying that this trend is only observed when one/both of these variables are varied would be informative.

L286 - I want to commend the authors on their clarity of communication in Sections 2.5 & 2.6, which I thought were really excellently written, and presented some very interesting results.

L367 - Some of the motivation for the quadratic form of this function should be given when the function is introduced, instead of in the discussion. Additionally, the assertion that the health burden of both the disease and the drug treatment scales super-linearly is rather bold, and should be supported by references to the wider literature - previous literature that I am aware of has tended to assume that the relationship between tumour size and patient quality of life is linear (https://doi.org/10.1002/ajmg.a.36466), while dose-limiting toxicity trials for daratumumab as a monotherapy (https://doi.org/10.1182/blood.2019000667) and in combination don't seem to show any support for a superlinear relationship (https://doi.org/10.1182/blood-2016-07-726729). If the quadratic form is required for numerical convergence of the continuous control problem then this is sufficient reason to use it (I am certainly not asking the authors to redo all the continuous control analysis with a different function!), but it should be clear that the quadratic form has been motivated by numerical restrictions rather than clinical applicability.
Finally, the assertion that double the drug produces more than double the harm presumably results from off-target drug effects, however I understood previously that the ODE model was formulated to include this explicitly instead of accounting for the harmful effects of excessive drug treatment through the cost function (as described in L143 & L290). This links to my previous comment about a lack of clarity over the 'cost of the drug', and I think may need rewriting across the multiple sections referenced above to provide clarity.

L620 - The doi given for reference 24 links to a Russian article - can the authors confirm whether they accessed the article in the original Russian, or include details of the translation they used?

L633 - The url is not line-broken within the margins of the page - while I recognise this is a formatting issue that would be fixed at publication, it does mean that the link doesn't work, making it harder to check the code.
Additionally, in the PLOS data availability statement (https://journals.plos.org/ploscompbiol/s/code-availability) it is recommended that authors specify details of their code licence and version of commercial software packages such as Matlab.

*Figure 1: Ideally the cell types would be labelled in the Figure/caption, so that the reader does not have to hunt through the text to find out what 'A', 'P' and 'N' are. The arrow colours are also difficult to see, especially for readers with colour-blindness.

Figure 6: Strictly speaking, the x axis in these plots is time not fitness. How about "The fitness of CD38- cancer cells decreases across the subplots (left to right)" or something

similar? Also the last sentence could benefit from additional clarification that the values quoted are the original values for CD38- cells, not CB38+ cells.

Finally, the authors should review the reference list, as there is inconsistent capitalisation (‘car-t’ vs ‘CAR-T’), missing capitalisation on proper nouns and incorrect quotation marks. Furthermore, the doi given for reference 24 links to a Russian article - can the authors confirm whether they accessed the article in the original Russian, or include details of the translation they used?

Authors should also review typographical errors throughout - here are examples highlighted from the author summary:
L21 - Common spelling is ‘abundant’ not ‘abundent’ in US English.
L26 - Common spelling is ‘resistant’ not ‘resistent’ in US English.
L27 - Incompatible subject-verb agreement with ‘prolongs’ and ‘increase’ - likely the latter should read ‘increase’
L28 - ‘costs’ -> ‘cost’
L29 - Common spelling is ‘intermittent’ not ‘intermittant’ in US English.

Further suggestions

The authors derive extensive optimal continuous and bang bang regimes - while direct comparisons under the current cost functions are not possible, would other comparisons between them be possible (i.e. are there circumstances where continuous or bang-bang control is preferable)? Furthermore, it would be interesting to discuss, in the conclusion, the possible experimental/clinical data needed to differentiate between these optimal treatment regimens (note that these data would not need to be integrated into this manuscript).

Building on this point, continuous control is typically not clinically feasible - however arguments that it is superior to bang bang control could motivate development of intermediate dosing levels. While I appreciate it may be out of the scope of this paper to consider a discrete N-level control (which acts as an intermediary between bang-bang and continuous control), it would be interesting to consider the analytic/computational feasibility of such an approach in the discussion, and talk about the applicability of traditional optimal control methods to this problem.

This could feed into wider comparison of continuous vs ‘bang-bang’ control methods - in what circumstances should one be preferred over the other? For example, the linear control function is shown in the final section to help identify mechanisms in the tumour model that affect the optimal control - is this an argument to use linear control instead of optimal control?
