# Supplementary material for: Optimal control of multiple myeloma assuming drug resistance and off-target effects
Source: PLoS Comput Biol. 2025 Aug 11;21(8):e1012225. doi: 10.1371/journal.pcbi.1012225 (PMC12364355; doi:10.1371/journal.pcbi.1012225)
Supplement: Attachment — Submitted filename: PCOMPBIOL-D-24-00948_DTW_review.docx [file pcbi.1012225.s004.docx]

The authors describe a mathematical model of multiple myeloma behavior under selection with the monoclonal antibody Daratumumab. They then use optimal control theory to derive optimal treatment schedules. The paper is well written and well described and represents a nice contribution to the cancer treatment optimization literature. I have a few suggestions for how to focus their analysis and make the results of their work easier to interpret but no major concerns with their methodology or key findings.

**Major Comments:**

1. It would be interesting to see the population dynamics under optimal control vs what they look like in the setting of treatment as usual like you see in the clinic. You could ignore pharmacokinetics and model it as a bang-bang treatment for a finite time or try and incorporate pharmacokinetics and model it as a continuous treatment with peaks in valleys. This would be useful both as a comparison to your formal optimization and as a sanity check on the way you have built and parameterized your model.
2. It might be worth adding some constraints to your OCT problem as an additional analysis – it is interesting that in a world unconstrained by maximum allowable dose/unit time the optimal solution just loads the system up with as much drug as possible and then stops. However, such a policy would not be feasible in real-world systems due to toxicity constraints, and even drug solubility concerns. There would also very likely be a maximum effective dose in this case due to all available CD38 receptors being occupied by inhibitor.
3. The authors consider the fitness decrement associated with CD38- cancer cells to be fixed. However, it is likely that persisters under drug selection undergo further evolution and ultimately become fully drug resistant (i.e. they can grow quickly in the presence of drug). The authors could consider modeling this evolution from drug tolerance to drug resistance by adding one additional compartment with small per time step transition probability.
4. The figure captions are too general and do not provide enough specific information about the information described to allow the figures to stand alone.
5. The figures all share the same design, which makes it a bit confusing to parse out what is actually different from one figure to the next, particularly when combined with the point above.

**Minor Comments:**

1. The authors place their work in the context of other methods that apply optimal control theory to mathematical models of cancer evolution. However, they would also be served by discussing related work on mathematical models of microbial evolution (Weaver et al in PNAS as one recent example). As a disclosure, I was involved with that study – feel free to just mine the references for relevant literature. There have also been several recent model-free treatment optimization approaches that leverage RL (**Gallagher et al, 2024 among many others**).
2. A few more lines in the intro describing Multiple Myeloma (i.e. it’s prevalence, mortality rate, likelihood of evading treatment, cost estimate for treatment if available) may help set the stage a bit better for the rest of the study.
3. Figure 1 would be easier to digest if the notation was defined either in the figure or in the caption (i.e. what do A, P, and N) refer to in the different compartments.
4. Related to above – more explicitly labeling the states in your results figures would be nice as readers won’t need to reference figure 1 or the methods to see what the notation means.
5. What does state refer to explicitly in your results figures? My brain assumes it is the proportion of the population that resides in each state but that doesn’t make sense because they don’t add up to one at any point.
6. Explicitly stating what alpha refers to somewhere in figure 4 would be a nice quality of life addition.
7. The authors should consider using more data rich representations for their sensitivity analyses – defining some summary statistic for control and then showing a heatmap for wide range of parameter values would give us a more deeper understanding of parameter regimes than the plotted trajectories.
8. Tense isn’t consistent in the discussion (past or present tense are used interchangeably.)
