# Supplementary material for: Optimal control of multiple myeloma assuming drug resistance and off-target effects
Source: PLoS Comput Biol. 2025 Aug 11;21(8):e1012225. doi: 10.1371/journal.pcbi.1012225 (PMC12364355; doi:10.1371/journal.pcbi.1012225)
Supplement: Attachment — Submitted filename: response_to_reviewers.docx [file pcbi.1012225.s005.docx]

All comments from reviewers have been combined in this document, together with the email from the editors, and are shown in black. Our responses are in red below the item or section we are responding to. Supporting quotes from the manuscript are in blue.

# Email from editor

7 October 2024

Dear Dr Lefevre,

Thank you very much for submitting your manuscript "Optimal control of Multiple Myeloma assuming drug evasion and off-target effects" for consideration at PLOS Computational Biology. As with all papers reviewed by the journal, your manuscript was reviewed by members of the editorial board and by several independent reviewers. In light of the reviews (below this email), we would like to invite the resubmission of a significantly-revised version that takes into account the reviewers' comments.

Three reviewers have now weighed in thoughtfully on this manuscript, and have come to very similar conclusions and concerns. While I am suggesting major revisions, I'd like to emphasize that the effort to improve the paper that the reviewers have expended is significant, and the revision must therefore be.

*In particular, the shared concern that this is purely theoretical model, and that the authors should make some attempt to contextualize their model (parameters) in either some inferred set from clinical studies, or potentially from experimental work.*

There also seems to be some shared concerns about the use of specific terms/definitions from optimal control theory. There remains within the work, however, meaningful novelty that was appreciated by all reviewers, for which the authors should be commended, and on which the decision to recommend revisions strongly rests.

We cannot make any decision about publication until we have seen the revised manuscript and your response to the reviewers' comments. Your revised manuscript is also likely to be sent to reviewers for further evaluation.

When you are ready to resubmit, please upload the following:

[1] A letter containing a detailed list of your responses to the review comments and a description of the changes you have made in the manuscript. Please note while forming your response, if your article is accepted, you may have the opportunity to make the peer review history publicly available. The record will include editor decision letters (with reviews) and your responses to reviewer comments. If eligible, we will contact you to opt in or out.

[2] Two versions of the revised manuscript: one with either highlights or tracked changes denoting where the text has been changed; the other a clean version (uploaded as the manuscript file).

Important additional instructions are given below your reviewer comments.

Please prepare and submit your revised manuscript within 60 days. If you anticipate any delay, please let us know the expected resubmission date by replying to this email. Please note that revised manuscripts received after the 60-day due date may require evaluation and peer review similar to newly submitted manuscripts.

Thank you again for your submission. We hope that our editorial process has been constructive so far, and we welcome your feedback at any time. Please don't hesitate to contact us if you have any questions or comments.

Sincerely,

Jacob Scott, MD

Academic Editor

PLOS Computational Biology

Pedro Mendes

Section Editor

PLOS Computational Biology

***********************

Dear Dr Scott and Dr Mendes,

Thank you for the opportunity to revise this manuscript. We appreciate the very careful and comprehensive reviews. Detailed responses to the reviewers’ comments are shown below.

Briefly, we have restructured the paper; a new supplementary section includes the original results section 2.4, the methods, and several new supplementary/sensitivity analyses requested by the reviewers. We have also significantly edited the paper. In particular, the introduction and discussion have been heavily rewritten and extended in response to the reviewers’ comments.

As per our correspondence, we did not aim to develop a complete, calibrated model of drug treatment for multiple myeloma that would be ready for clinical application. Rather, our modelling effort is quite theoretical, focused on a particular mechanism of drug evasion and its consequences for optimal drug treatment. We have now specifically included this point in the final paragraph of the discussion. The summary comments from the first two reviewers indicate that they understood this limitation and still found significant value in the paper.

While we were unable to fully respond to some concerns of Reviewer 3, we have attempted to improve the framing and biological context. Overall, we believe we have been able to provide a strong response in a large majority of cases.

We thank the reviewers for their detailed and insightful critiques. We believe that the manuscript has been greatly improved in response. We hope it is now acceptable for publication but would be willing to make more changes if needed.

Yours sincerely,

James Lefevre

# Reviewer 1

Optimal control of Multiple Myeloma assuming

drug evasion and off-target effects

Summary

The authors proposed to use optimal control techniques to derive treatment schedules, within the disease context of multiple myeloma. This was conducted on an ODE model of a partially drug resistant tumour, building on previous work by Khalili et al. and Sharp et al. (who are cited in the manuscript). Novel to this paper, the authors also allowed for both drug evasion (via reversible CD38 expression) and off-target killing of healthy cells.

The inclusion of these additional phenomena is well motivated within the study. It is worth noting that the study is purely theoretical (neither the ODE model nor the derived treatment regimens are parameterised by nor compared to experimental/clinical data), however the authors do well to convey those limitations. The authors also provide code for their model, although I have detailed difficulties in running this below.

These additional drug dynamics motivate quantitative (i.e. extended treatment times in Sections 2.2 & 2.3) and qualitative changes (Sections 2.5 & 2.6) to the optimal treatment approach. The latter are particularly exciting, demonstrating that optimal binary dosing strategies may consist of either single or multiple treatment periods depending on the parameter regime. Within the context of this class of models, this appears to be the first demonstration of optimal cyclic controls.

However, the authors employ an unconventional form of optimal control, that I believe is mis-categorised in the paper as ‘bang-bang’ control; I provide more details of prior approaches to cancer therapeutic scheduling using conventional ‘bang-bang’ control below. I recommend that the authors reframe the narrative of this paper to focus on the impact of different objective cost functions (within a continuous control framework). This would build upon established literature in the field, and I believe would also present their current results through a stronger narrative which could be of wider interest.

We thank the reviewer for their very thorough review. We have substantially revised and extended our paper in response to their recommendations. Our detailed responses to their concerns are given below.

## Significant Revisions

I have split the body of this review into more significant, and minor, revisions. While I appreciate the review is lengthy, I hope that this will make the content more digestible, and I firmly believe it will strengthen this interesting paper. Additionally, I have endeavoured to signify minor revisions that may require (small) additional analysis or visualisation changes by ‘**’ and ‘*’ respectively, with other revisions relating purely to the text itself. I hope that this will support the authors when revising this manuscript.

In general, this paper would benefit from further context of previous work applying optimal control to immune/targeted therapies, and additional clarity on the novel contributions of this paper relative to previous work in the field. In particular, I would like to draw the authors attention to recent work by Khailov et al. (https://doi.org/10.3390/g11040053) that builds on the cited work by Grigorenko et al. and applies optimal control to a Leukaemia treatment model. They identify different regimes in which continuous or intermittent therapies (under bang-bang control) are optimal, akin to the results in the final results of the submitted manuscript (though these results only consider single breaks in treatment, rather than the cycling behaviour explored by the authors). More recently, Ledzewicz et al. (https://doi.org/10.3934/dcdsb.2023141) have integrated a similar approach with the relative weighting approach I mention below. The authors may find it beneficial to compare with these approaches to bang bang control, which avoids the problem of intermediary doses that the authors experienced (though is not without its own drawbacks).

In this vein, my primary concern is that the formulation of ‘bang-bang’ control in this paper does not seem to match with existing literature, such as traditional formulations (https://doi.org/10.1016/0022-247X(63)90081-7, https://www.jstor.org/stable/43634288), and prior literature applying conventional bang-bang control to chemotherapeutic scheduling (https://doi.org/10.1023/A:1016027113579). I believe this is the cause for the intermediary control values observed in Sections 2.4 onwards.

I therefore have to disagree with the final paragraph of Section 2.4 - to my understanding this behaviour is not typically present in bang-bang control because bang-bang control is traditionally formulated to prevent this behaviour (the control is assigned according to the sign of the switching function - see refs above). Because these results could not be replicated by a conventional method of bang-bang control, it seems to me that they are necessarily an artefact of the method used here. Indeed, the conventional formulation of ‘bang-bang’ control, wherein the control is determined by a switching function, has also been adopted by Sharp et al. (whose work is cited in this paper as the basis for the authors’ approach). This work appears to avoid the issue of non-binary controls above - while I see this may be more challenging to formulate for the cycling solutions in Section 2.6, it would certainly help the convergence issues in previous sections.

I should emphasise that I do not think it is necessary for the authors to reformulate this control method, though the authors may pursue this if they wish. However, it may be more accurate to describe this as a different form of continuous control, with a linear control function designed to favour (but not limited to) extremal values. I believe this would offer a compelling overall narrative, comparing the impact of different cost functions instead of different control methods to show that this qualitatively changes the forms of the derived control. For some context on prior work addressing this question, I recommend https://doi.org/10.1007/s10957-020-01754-2; <https://doi.org/10.1016/j.mbs.2010.11.007>.

We thank the reviewer for this important clarification, and for the linked papers; we now cite almost all of these papers. The 2020 survey by Ledzewicz et al. provided particularly a useful discussion of cost functions and control forms.

We no longer incorrectly use the term “bang-bang” control; instead, we refer to the two cost functions. We have also adopted the “bang-singular” terminology recommended by Reviewer 3. We have added some relevant discussion to the Introduction and Results.

In Section 1.3 (cost functions) we have removed all reference to the bang-bang and continuous control forms, and restricted the discussion to the cost functions.

In Section 2.2, after presenting the optimal controls for the default model, we have added the following text:

Linear costs typically result in optimal control levels at either the maximum level or zero. In the Null-N model all solutions consisted of an initial period at maximum level followed by an abrupt and final end of treatment. This is known as a bang-bang control, although this term may refer to controls that are explicitly constrained to either a maximum or minimal value at each time ([34, 35], or more recent work such as [36]). In our model, however, the linear cost function produces an initial period at maximum level followed by a period of intermediate level control. This intermediate period is known as a singular arc, and the control is a concatenation of bang and singular controls which we refer to as bang-singular [32].

Section 2.4 has been moved to the supplementary section (Section S2.1) as recommended elsewhere, and we have deleted the final paragraph of this section.

The Discussion has been heavily rewritten and extended, with the advice above taken into account.

In particular, the authors may wish to comment on the generalisability of this approach to other cancer sites or therapeutic agents in the discussion. It might be interesting to note that some drugs are conventionally approved at multiple dosing levels (or even across a dosing spectrum); the authors can therefore argue that the cost function could be modified based on the drug context, with quadratic functional forms being more appropriate to drugs with multiple dosing levels than linear forms.

This would also feed into the authors’ wider narrative on the functional forms of the cost function. I think this is a very interesting area that is not done justice in the current manuscript; the cost functions are introduced with minimal explanation for their functional form in Section 1.3. The functional forms are commented on further in Section 2.2, explaining that the linear form is required for the algorithm to favour binary dosing, but this doesn’t explain the quadratic form of the drug cost, given it is criticised in L218, and later in L374. While I have no qualms with the use of a quadratic cost function given its importance within the optimal control literature, the authors should be explicit whether this is motivated by any real-world application or simply a mathematical requirement for the optimal control framework. This will help reconcile the use of this approach with the authors’ own recognition that this cost function differs significantly from reality, and help persuade the reader that a control solution (treatment schedule) that minimises the square of the daily drug cost (instead of the cumulative drug cost) has real-world relevance.

Additional comparison of the cost functions used would also be helpful - both linear and quadratic cost functions are used in this study, but the circumstances under which each method should be preferred are not clear. Perhaps linear cost functions are more appropriate when binary dosing treatment schedules are desired (due to clinical restrictions?) The authors could also argue that the linear dose function has helped them identify mechanisms within the ODE model that affect the optimal control, whereas this would not have been possible with the quadratic function. This could also extend the applicability of this work to other cancers/biological systems outside of multiple myeloma, if the linear cost function is promoted as a method to identify which mechanisms within a complex model affect the model’s optimal control.

We have reworded the relevant sections extensively. In Section 1.3 (Introduction / cost functions) our justification of the quadratic cost still rests on the uncertainty of the true costs and the goal of checking the robustness of trends under different cost assumptions, but we now explicitly reference the popularity of the quadratic function in the literature and its numerical/mathematical advantages:

Quadratic functions are also widely used, with the justification that super-linear costs are evident in many contexts. This may also be due in large part to their superior mathematical and numerical tractability compared with linear functions [32].

A paragraph of the Discussion is now devoted to a more detailed and consolidated reflection on the quadratic cost function, in which we reiterate its value as a test of cost function sensitivity, but also its limitations and our preference for the linear cost results. Further discussion, mostly new, considers qualitative interpretation of the linear cost controls alongside clinical considerations (acknowledging the limitations of biological fidelity), concluding with:

Nevertheless, our demonstrated capacity to identify the model dynamics that favour extended treatment at reduced intensity, or more continuous vs discrete treatment, has potential to inform treatment regimes. In contrast, the quadratic cost controls we obtained lacked this qualitative information, while linear cost controls of an enforced bang-bang form would be less informative given the availability of multiple Dara dosing levels.

In further discussion of the cost function, the authors recognise that the correct weighting between the health cost of the cancer and the financial cost of the drug is not obvious. However they then sum these two costs with no additional scaling, in effect assuming a very specific weighting between these two costs. While I recognise that this would be a major change, modifying the cost functions to include a tunable parameter that scales the relative weighting of these two costs (i.e. by replacing the drug cost ‘u’ with ‘ beta u’ for some unknown beta) would generate significantly more generalisable solutions - see Sharp et al. for an example of this approach. The authors could then verify that their qualitative conclusions are not dependent on the value of beta that they have implicitly assumed (either through a systematic evaluation of the system when beta is varied, or simply by selecting a discrete number of values of beta to test). At the very least, I think that the authors need to be explicit that an implicit balance between these two costs has been chosen here, though I do feel that that reduces the strength of the paper’s conclusions somewhat.

Although our code allows for variation in the weighting, we did not explore this in our original analysis due to the number of factors already under consideration. Instead, we chose parameters such that the drug and cancer terms of the cost functions would have comparable magnitude.

However, we agree that the cost weighting is important to consider. Hence, we performed a supplementary analysis (Section S2.3) in which we explored a two-fold change in the weight ratio in both directions. This subsection includes notes on the small modification to the methods required for reweighting. We confirmed a comparable contribution between the cost function terms, and found a minimal change in controls under this variation. This supplementary analysis also includes a brief look at blended linear/quadratic cost. Based on this supplementary analysis demonstrating the robustness of our results to reasonable variations in cost weighting, we determined that our original approach was appropriate and therefore maintained our unweighted formulation in the primary analysis.

We have added the following clarification in the Introduction (Section 1.3):

It is reasonable to ask whether different weightings affect the form of the optimal control (see [33], for example), and in Section S2.3 we perform a sensitivity analysis in which we consider altered weights.

In addition to these comments on the cost function, I have a few other significant suggestions for the paper, particularly in mind of the typical style and format of papers in PLOS Computational Biology, and the connection to biological systems.

I found Section 2.4 very confused - in comparison to Sections 2.5 & 2.6 which I thought were excellently written, I wasn’t clear on the salient points from this section. I have included some suggestions in the line-by-line comments below on how to address this and simplify that section, but I wondered whether this section might be better placed in the Supplementary Information? This might also mitigate a confusing section of the discussion (L347-350), where the authors mention the mixed results from Section 2.4 but struggle to motivate or explain them further. I have provided further suggestions on this section (including the figures) in minor points below.

The reviewer makes a good point, and we have moved Section 2.4 to the supplementary material (Section S2.1). We have also modified the discussion; the results of this section are reviewed in the fourth paragraph, but are otherwise de-emphasised.

I also suggest that the authors review their figure captions; I think it would be helpful for the figure captions to highlight the important trends that a reader should be looking for in these figures in addition to simply labelling the figure. For example the caption for Figure 4 simply states these are optimal control solutions, but could also draw the reader’s attention to the reduced drug duration under both control methods as immunity is strengthened (left to right).

It would also be helpful to remind the reader what each parameter refers to in these captions (throughout the paper), particularly those that are crucial to understanding of the figure (such as those varied between subplots), so the reader does not need to refer back to the original table - authors could label figures/axes/subpanels with the variable names (with the symbols in brackets) and indicate how model behaviour changes between subpanels descriptively (i.e. ‘Increasing Immunity Strength’ on an arrow going left -> right in Figure 2a).

We have extensively modified figure captions as suggested.

Furthermore, while I felt that the limitations that the model assumptions placed on the applicability of this work were well described, I detail below some of the methodological decisions that also seem to limit the applicability of this paper to real-world systems, and would benefit from further explanation/justification. In particular, in Section 2.6, it seems drastic to remove the off-target effect completely (L331), especially as the authors argue previously that this is an important biological effect to include. Can the authors show that the cyclic solution vanishes when the off-target effect is restored to reasonable levels (such as $\mu_{Au} = 0.1$, instead of setting $\mu_{Au}$ to zero? Alternatively if the cyclic solution only re-emerges when $\mu_{Au} = 0$ then this should be stated.

We have extended this analysis to include the default value *µ_Au_* = 0.1 as well as zero, to help close this gap in the analysis. The cyclic behaviour is lost in both cases.

Finally, the authors have also provided code with their manuscript. I have been able to verify that the simulation examples script runs without error, and replicated results from Figures 2.

However I encountered issues with the optimal_control_example file, and was only able to replicate Figure 3 as well as some panels from Figures 9. Matlab repeatedly threw errors when saving a png version of the figure generated (such as in L69). This is attributed to an invalid figure handle (I suspect due to a terminated simulation being handled internally), and I recommend the authors investigate this further. I was using 64-bit Matlab R2023a (9.14.0.2206163).

We believe this was due to the change in behaviour of the implicit figure and axis handles gcf and gca in the more recent versions of Matlab. These have been removed from all code, and replaced with explicit handles. We have also added the code used for new analyses, with examples.

## Minor Revisions

As mentioned above, revisions that may require (small) additional analysis or visualisation changes are denoted by ‘**’ and ‘*’ respectively, with other revisions relating purely to the text itself.

L13 - The phrase ‘we identify a general increase in treatment duration and costs’ is unclear, and would benefit from further context - what change has resulted in this increase? Or does this statement refer to an observation that optimal strategies typically have longer treatment duration and higher costs (in which case the text could be updated to reflect this)?

This sentence has been modified for clarity. We now say (new text underlined):

We identify a general increase in the duration and costs of optimal treatment, as a result of these added mechanisms.

L45 - From a clinical perspective, it would be worth clarifying whether mono-therapeutic Daratumumab is typically applied as a first line therapy, or later in treatment (to tumours that have already been exposed to multiple drugs and potentially metastasised). Similarly, it would be helpful to explain whether Daratumumab is only used continuously in the clinic, or whether intermittent treatment regimes have already been explored clinically?

We have extended the background on multiple myeloma and its treatment at the start of the Introduction, including the following text:

More recently combination therapies using Dara have also been developed [11]. However, in this work we consider treatment using Dara only. Dara monotherapy has primarily been used for heavily pretreated relapsed or refractory disease [5], although it has shown efficacy in preventing the precursor condition, smoldering multiple myeloma, from progressing [12].

In the Discussion we now discuss our results in relation with clinical practice, including the text:

In clinical practice, Dara treatment consists of discrete multi-hour infusions, typically repeated at weekly or longer intervals, with multiple dosing levels used [40].

L46 - The authors switch between the terms ‘tolerance’, ‘resistance’ and ‘avoidance’ somewhat interchangeably - to be more in line with cancer literature I would advise using the term ‘resistance’ (e.g. see <https://doi.org/10.1038/nrmicro.2016.34>).

We now use ‘resistance’ throughout the document, including the title. We have also replaced ‘evades’ with ‘resists’.

L49 - Terminology such as ‘occur passively’ is used in a slightly vague/confusing manner. The authors could do more to differentiate between Darwinian evolution (changes in species abundance through means of natural selection), and Lamarckian evolution (individuals actively adapting to the environment, through mechanisms such as drug-induced switching, and passing the changes on to off-spring).

We have removed the terminology ‘passively’, and instead make the distinction between Darwinian and Lemarckian evolution.

L53 - It is not clear which off-target effects are being referred to here.

We now say (new text underlined):

Using optimal control theory, we investigate how this drug resistance affects treatment protocols for Dara that balance the cost of treatment with the burden of disease. The cost of treatment includes health impacts as well as the financial cost of the drug and its administration. We also include an off-target effect in our model, in which the drug causes mortality in the surrounding population of healthy cells.

L54 (and elsewhere in introduction) - If the authors are referring specifically to the financial cost of treatment here, then it might be helpful to clarify this (or otherwise to explain which costs they consider within scope for this paper).

We have added the following clarification in the Introduction:

The cost of treatment includes health impacts as well as the financial cost of the drug and its administration.

L62 - The topic of maintenance treatment has not really been addressed previously in this paper, and is not discussed again in the paper. If the authors wish to retain this reference, more explanation of this treatment context would be helpful.

We have added the following explanation (new text underlined):

This may help to inform maintenance Dara treatment, which has shown success preventing relapse when used as a reduced frequency monotherapy following the primary combination therapy [15].

L64 - This introduction explains optimal control very well, but is likely too general for the intended audience, and could be greatly shortened.

We have removed much of the more basic dynamical systems and control introduction (original lines 65 to mid 72, and most of lines 90-96).

L100 - I wish to commend the authors on the labelling of equation (3), which aids the clarity of the text significantly.

Thank you.

L128 - The authors should clarify what data was used to calibrate the model described here, as the cited papers do not give detail on this.

The word “calibrated” was inappropriate and has been removed. As noted in the final sentence of Section 1.2, the Sharp model was not calibrated to real data.

**L135 - Could the authors indicate the timescale of this divergence? Given this system is not necessarily assumed to reach steady state, transitory effects may well be important.

This divergence permanently closes as the upstream population converges to its steady state. Our calculated optimal controls start from steady state, so there will be no impact on our results. We have added the following clarification:

There is an implicit assumption that the stem cell population is at steady state; this causes only a transitory divergence from the Sharp model, and has no impact on the optimal control results as they assume steady state at *t* = 0.

L141 - The authors assume that ‘fitness is significantly lower in N’, however it is not clear how this manifests in the model - additional clarity is needed on what parameter values are affected by this assumption. I assume this is the reasoning for $\rho_{P} > \rho_{N}$? And is there any evidence for this fitness cost?

We have noted that the loss of fitness affects both death and proliferation, but we have not stated the parameters as they are not introduced until the equations and table below. This is an assumption, but we have added support from another cancer system:

It has been shown that CD38 is a growth and survival factor in a lung cancer model system [5], and so it is reasonable to assume that *N* has both lower proliferation and higher death rate than *P*.

L143 - This is an excellent point, and indeed a key differentiation between the authors’ work and Sharp et al. ([27]) - I think that further explanation of why this interaction with population dynamics is beneficial would strengthen this paper.

We have added the following explanation:

…; in particular, a depleted healthy population may reduce space competition and allow the cancer population to recover faster.

L145 - The state variable A has not been defined at this point in the text (and I believe is only ever defined implicitly in Table 1) - a more explicit definition in the model description would be helpful.

We now introduce *A* earlier alongside *β_A_* as follows (new text underlined):

The upstream stem cell population is modelled implicitly as an influx of healthy cells into the marrow (rate *β_A_*), supporting the healthy marrow population *A*.

L150 - The inclusion of a complete table of parameter values is much appreciated! It would be helpful to indicate the providence of these values, so the reader can assess which values were taken from the Sharp model and which were defined by the authors. Given some values have been modified from the Sharp model, perhaps that process could be explained in Supplementary Information/Methods - for example it is not clear how the drug killing parameter values were updated, as drug is assumed to only affect stem cells in the Sharp model, while the authors do not model stem cells explicitly.

Additionally, I could not find numerical values for the initial population sizes used – perhaps they could also be included in this table?

Finally, I found that description “Rate of loss in CB38 expression in P” slightly confusing (as this implied to me that this is an internal transition within the P state) - perhaps the authors could consider rephrasing to “Rate of loss of CB38 expression from P (to N)” or something akin to this?

We have added an extra column for the Null-N model parameters in the table, and added explanation of parameter providence as follows (new text underlined):

A description of the model parameters, and the default values used, are listed in Table 1. In the column on the right hand side we list a second set of parameter values which, together with the condition *N*(0), define the *Null-N* model. This simplification reproduces the core features of the Sharp model and is used as a negative control.

The non-zero parameters of the Null-N model were adapted from the Sharp model (Table 1 of [31]), which were selected to produce balanced dynamics supporting both healthy and cancerous states and the capacity for effective drug control. Our parameters *µ_A_*, *ρ_P_* and *µ_P_* correspond to *δ_A_*, *ρ_L_* and *δ_L_* respectively in the Sharp model, *β_A_* = *δ_S_*(1 – *δ_S_*/*ρ_S_*) is the exit rate from the *S* compartment at steady state, and the remaining parameters retain the same name. These values are also used in the full model, except for a small adjustment to *ρ_P_* and *µ_P_* to balance the introduction of *N* with substantially lower proliferation and higher mortality. The additional parameters in the full model were described previously, other than the Dara related parameters which we consider now.

We have also rephrased the expression switching descriptions as suggested.

L172 - Additional justification is required for the removal of the final state cost, and particularly the assertion that this results in more tractable computations - can the authors provide a reference for this statement?

The assertion that *ϕ* = 0 is "generally preferred as it provides more tractable computations" was poorly considered and has been removed. However, we have not been able to formulate terminal costs which reasonably represent the costs of ongoing treatment; the long-term cost of a very small amount of residual cancer may vary dramatically depending on whether the cancer cell population will expand again without treatment, and what level of treatment is required to prevent or limit such a recurrence. Instead, we have added a supplementary analysis (Section S2.4) to address this issue, together with related concerns raised by Reviewer 3 (major concerns section 3) around extrapolation of finite time-window results to an infinite horizon context.

The final paragraph of the cost function subsection of the introduction has been rewritten to reflect the points above, and to reference the supplementary analysis. The results section has been edited at several points based on the findings of this analysis. We have also added a substantial paragraph on methodology in the discussion, which includes this issue.

*L190 - Particularly in Figs 2.b and 2.f, it does not appear that the A and P populations have reached steady state - while it seems plausible that they might in the near future it is also not implausible that A might decrease to extinction if simulated for sufficiently long. If the authors wish to state that these populations have reached a balance, I think it would be necessary to show a longer timescale on these plots, as currently they don’t seem to agree with the text.

We have increased the end time from 50 to 150.

*L196 - Similarly, Fig 2.h doesn't provide any visual evidence for the claimed reduction in drug efficacy compared to Fig 2.d. While the N population here is non-zero, there is no visually perceptible impact on the A/P populations, nor the timescale over which the cancer (P+N) is eliminated.

The reviewer makes a valid point. To avoid this visually unsupported implication, we now say:

In the presence of a high dose drug control (g), the role of this population grows and has the effect of diminishing drug efficacy via the escape mechanism. Panel (h), however, shows little or no difference in drug efficacy from (d), suggesting that the drug resistance mechanism plays a larger role when the control has higher intensity and shorter duration;

L199 - This begs the question, why include the off-target effect in the first place? I think the authors address this very well in Section 2.5+, but should signpost that section here, so that the reader can easily see why this effect was included in your model.

We have signposted these later sections by adding the following sentence at the end of the paragraph (note that 2.4 and 2.5 are the original 2.5 and 2.6):

However, in Sections 2.4 and 2.5 we consider higher values and see important effects.

L224 - I am not clear why this provides support for extended treatment? It seems more intuitive to me that this would provide support for applying continuous control over bang-bang control, as a cost function designed to favour binary dosing (u = 0 or 1) has instead selected an intermediary value.

We trust that this is clearer after fixing the use of “bang-bang” terminology. The solution is in fact a continuous control (at least piecewise). We are trying to convey that the linear cost does not create any bias towards smoothing or stretching out the treatment, so the form of the solution must reflect the model dynamics.

L232 - It isn’t clear what the authors mean by burden in this context - the tumour burden is typically used to refer to the number of cancer cells/amount of cancer cells in the body. In that sense, the burden of N cells is decreasing here - but perhaps the “role” of N cells is significant as they can persist under treatment, resulting in a tumour that cannot be eliminated. This would then agree with the excellent later explanation given around L240.

We have rephrased this as follows:

… this is associated with the majority of the cancer cell population becoming CD38 negative.

L248 - I am somewhat confused by this point - it seems that the authors are referring to a common phenomenon in ODE models wherein a cell population may decay exponentially, but cannot be fully eliminated due to the continuous nature of the model. While this flaw of the Null-N model is not catastrophic to its biological realism in my view, I recognise that it does pose issues for optimal control. However, to argue that the author’s model is superior would require them to show that their model does not exhibit this behaviour - while this may indeed be the case, I can’t see it in this section. Perhaps the authors could make a more explicit comparison between the problematic behaviour they see in the two models, and provide a more detailed explanation of how this behaviour practically affects the biological realism of the models.

We have added the following sentence of explanation:

In contrast, cancer populations in the steady state treatment regime in Fig. 4 remain non-negligible, and recurrence does not depend on exponential increase from an arbitrarily low level.

*L260 - I did not find the b panels very helpful in Figures 5-7. I would recommend that the authors reconsider whether these are essential to the main narrative, or could perhaps be removed/moved to the supplementary information? If the authors do wish to retain these subpanels in the main text, I think that additional description is necessary to explain the novel results/insights presented in these subpanels, that could not be inferred from the other subpanels.

This could also help towards condensing Figures 5-7, as they currently comprise of 22 subpanels which seems gratuitous for their role in the paper, and makes them harder to interpret. For example, from the 8 panels in Fig5a, the only trend I can see (and is commented on in the text) is that the transition from u=1 to u=0 is extended as the switching rate P <-> N increases, which could be conveyed in a single panel depicting these four curves for u. This would help isolate and emphasise this important result, and also provide space for a second subpanel displaying the trend in aggregate control, which the authors mention in the text but do not show.

All of Section 2.4, including Figures 5 to 7, has been moved to the supplementary material (Section S2.1). In Section 2.5 (originally 2.6) we reference this briefly and note two points of interest. This includes the text:

We note that the rate of expression switching appears to influence the shape of the linear cost control, while the drug-induced loss of CD38 expression has a strong influence on the prolongation of treatment.

**L265 - Figure 5 displays some counterintuitive results, which I think warrant further explanation. We see an extended time at intermediate dosing levels, which the authors claim results in a higher aggregate drug level despite the shorter initial duration of maximal drug (aggregate drug levels are not shown though this might be helpful). Yet the aggregate control is not (visually at least) raised under continuous control - I can appreciate that the two control methods will distribute drug in different ways, but why does one method need more drug to cope with increased P <-> N switching whereas both the magnitude and timing of drug is completely unaffected under the other control? We also see this trend in Bang-bang control in Figs 6/7, but there the drug profile in Continuous control is also modified accordingly.

This is an interesting point – it does seem to be the only case where the change in the linear control is not reflected at all in the quadratic control. We moved the initial sentence noting minimal change in the quadratic control to the end of the paragraph, and expanded to the following:

Notably, there is almost no corresponding effect on the quadratic cost optimal control, in contrast to all other parameter variations considered (Figures 4, 5, S2, S3). This is likely related to the nature of the change in the linear controls; increasing the switching rates does not greatly increase the length of treatment, and the tapered shapes of the quadratic controls already include a similar period of control at reduced intensity.

We considered additional analysis to gain a clearer understanding of this, but decided against since this material is now supplementary, and does not bear strongly on the main results. Note that the initial and aggregate drug levels for the linear cost control are shown in panel (b).

**L273 - From a point of interest, has any attempt been made to disentangle these effects? Presumably it would be fairly quick to rerun this analysis while holding either proliferation or mortality constant - the addition of even a short comment saying that this trend is only observed when one/both of these variables are varied would be informative.

We have not attempted to disentangle these results. This would be a reasonable additional analysis but given that this material is now in the supplementary section and relative fitness is a peripheral issue, we decided against pursuing this.

L286 - I want to commend the authors on their clarity of communication in Sections 2.5 & 2.6, which I thought were really excellently written, and presented some very interesting results.

Thank you!

L367 - Some of the motivation for the quadratic form of this function should be given when the function is introduced, instead of in the discussion. Additionally, the assertion that the health burden of both the disease and the drug treatment scales super-linearly is rather bold, and should be supported by references to the wider literature - previous literature that I am aware of has tended to assume that the relationship between tumour size and patient quality of life is linear (https://doi.org/10.1002/ajmg.a.36466), while dose-limiting toxicity trials for daratumumab as a monotherapy (https://doi.org/10.1182/blood.2019000667) and in combination don’t seem to show any support for a superlinear relationship (https://doi.org/10.1182/blood-2016-07-726729). If the quadratic form is required for numerical convergence of the continuous control problem then this is sufficient reason to use it (I am certainly not asking the authors to redo all the continuous control analysis with a different function!), but it should be clear that the quadratic form has been motivated by numerical restrictions rather than clinical applicability.

Finally, the assertion that double the drug produces more than double the harm presumably results from off-target drug effects, however I understood previously that the ODE model was formulated to include this explicitly instead of accounting for the harmful effects of excessive drug treatment through the cost function (as described in L143 & L290). This links to my previous comment about a lack of clarity over the ‘cost of the drug’, and I think may need rewriting across the multiple sections referenced above to provide clarity.

The cost function section of the introduction (Section 1.3) has been substantially rewritten and now includes the following justification for the cost function choices:

In the absence of specific information, a simple linear combination of the cost factors is the most natural choice for the cost function. Quadratic functions are also widely used, with the justification that super-linear costs are evident in many contexts. This may also be due in large part to their superior mathematical and numerical tractability compared with linear functions [32]. We consider two optimal control cost functions, linear and quadratic in form (7, 8), to provide insight into the influence of the cost assumptions and the robustness of any conclusions.

Lines 216-217 in the results have been reworded to:

However, if the true costs include any linear component such as the financial cost of drug supply, the quadratic cost function will be least accurate at lower levels of control and cancer. Hence the very low cost associated with an extended period of low-level treatment may not be realistic.

We have substantially revised the discussion based on these points and the related comments of Reviewer 3 (major concerns, section 3). We cannot find support for super-linear costs in this system and this is reflected in the revision:

Although the quadratic cost function results helped to demonstrate the robustness of many of the trends we identified, we conclude that these controls are of less interest than the linear cost controls, notwithstanding the greater ease of computation. Indeed, the literature provides little support for a super-linear health burden of either cancer [37] or Dara [38, 39]. Even if super-linear costs do exist, the very low cost assigned to an extended period of low dose treatment is unlikely to be realistic; if real costs include at least some linear factors, such as the financial drug cost, these will tend to dominate as *u* and *P*+*N* approach zero.

L620 - The doi given for reference 24 links to a Russian article - can the authors confirm whether they accessed the article in the original Russian, or include details of the translation they used?

We could not find a translation for reference 24. While the English abstract plus the figures and equations sufficiently support the citation, it was a poor choice. We have replaced it with your suggestion by Khailov et al. that builds on it.

L633 - The url is not line-broken within the margins of the page - while I recognise this is a formatting issue that would be fixed at publication, it does mean that the link doesn’t work, making it harder to check the code.

We have added an MIT license to the code, and added this information and the Matlab version to the PLOS data availability statement. We have also added a line break to avoid splitting the url.

*Figure 1: Ideally the cell types would be labelled in the Figure/caption, so that the reader does not have to hunt through the text to find out what ‘A’, ‘P’ and ‘N’ are. The arrow colours are also difficult to see, especially for readers with colour-blindness.

We have added cell types to the caption. We now use green, purple, and black for the arrow colours, and we have thickened the lines to improve legibility.

Figure 6: Strictly speaking, the x axis in these plots is time not fitness. How about “The fitness of CD38- cancer cells decreases across the subplots (left to right)” or something similar? Also the last sentence could benefit from additional clarification that the values quoted are the original values for CD38- cells, not CB38+ cells.

The caption to this figure (now labelled Figure S2) has been changed to:

Effect of CD38- myeloma (*N*) fitness on optimal treatment: optimal control solutions for a range of *ρ_N_* and *µ_N_* values. The fitness of CD38- cancer cells decreases across the subplots (left to right), with the left most value representing fitness equal to the CD38+ cells (original values for CD38- cells *ρ_N_* = 0.15 and *µ_N_* = 0.06). For both cost functions, increased *N* fitness corresponds to more prolonged treatment and higher overall costs, especially as fitness reaches the same level as *P*.

Finally, the authors should review the reference list, as there is inconsistent capitalisation (‘car-t’ vs ‘CAR-T’), missing capitalisation on proper nouns and incorrect quotation marks.

Furthermore, the doi given for reference 24 links to a Russian article - can the authors confirm whether they accessed the article in the original Russian, or include details of the translation they used?

We have fixed these issues. Further, as mentioned previously, we have replaced reference [24] with the paper by Khailov et al.

Authors should also review typographical errors throughout - here are examples highlighted from the author summary:

L21 - Common spelling is ‘abundant’ not ‘abundent’ in US English.

L26 - Common spelling is ‘resistant’ not ‘resistent’ in US English.

L27 - Incompatible subject-verb agreement with ‘prolongs’ and ‘increase’ - likely the latter should read ‘increase’

L28 - ‘costs’ -> ‘cost’

L29 - Common spelling is ‘intermittent’ not ‘intermittant’ in US English.

Thank you for alerting us to these errors, which have now been corrected.

## Further suggestions

The authors derive extensive optimal continuous and bang bang regimes - while direct comparisons under the current cost functions are not possible, would other comparisons between them be possible (i.e. are there circumstances where continuous or bang-bang control is preferable)? Furthermore, it would be interesting to discuss, in the conclusion, the possible experimental/clinical data needed to differentiate between these optimal treatment regimens (note that these data would not need to be integrated into this manuscript).

Building on this point, continuous control is typically not clinically feasible – however arguments that it is superior to bang bang control could motivate development of intermediate dosing levels. While I appreciate it may be out of the scope of this paper to consider a discrete N-level control (which acts as an intermediary between bang-bang and continuous control), it would be interesting to consider the analytic/computational feasibility of such an approach in the discussion, and talk about the applicability of traditional optimal control methods to this problem.

This could feed into wider comparison of continuous vs ‘bang-bang’ control methods – in what circumstances should one be preferred over the other? For example, the linear control function is shown in the final section to help identify mechanisms in the tumour model that affect the optimal control - is this an argument to use linear control instead of optimal control?

The discussion has been expanded to touch on many of these points.

# Reviewer 2

The authors describe a mathematical model of multiple myeloma behavior under selection with the monoclonal antibody Daratumumab. They then use optimal control theory to derive optimal treatment schedules. The paper is well written and well described and represents a nice contribution to the cancer treatment optimization literature. I have a few suggestions for how to focus their analysis and make the results of their work easier to interpret but no major concerns with their methodology or key findings.

## Major Comments:

1. It would be interesting to see the population dynamics under optimal control vs what they look like in the setting of treatment as usual like you see in the clinic. You could ignore pharmacokinetics and model it as a bang-bang treatment for a finite time or try and incorporate pharmacokinetics and model it as a continuous treatment with peaks in valleys. This would be useful both as a comparison to your formal optimization and as a sanity check on the way you have built and parameterized your model.

We agree that this would be interesting, but given the lack of model calibration against real data we could not devise a sufficiently meaningful way of doing this.

2. It might be worth adding some constraints to your OCT problem as an additional analysis – it is interesting that in a world unconstrained by maximum allowable dose/unit time the optimal solution just loads the system up with as much drug as possible and then stops. However, such a policy would not be feasible in real-world systems due to toxicity constraints, and even drug solubility concerns. There would also very likely be a maximum effective dose in this case due to all available CD38 receptors being occupied by inhibitor.

The upper bound on *u* for the linear cost function is naturally interpreted as either the maximum effective dose (due to receptors and so on) or any limits imposed by toxicity etc, whichever is most restrictive (although obviously an approximation as we ignore pharmacokinetics). The quadratic cost lacks a hard upper bound, although we note that it effectively limits the dose to within the bounds imposed on the linear cost controls.

We considered examining the effect of varying the upper bound. This would be a reasonable variation to consider for the linear cost control. But if we performed a parallel analysis using the quadratic cost, interpretation would be difficult, as the hard bound would only have an effect in some cases and it is unclear that comparing between the linear and quadratic cost cases would make sense. Ultimately, given the manuscript length, we decided against extending the scope of the paper in a way that is not strongly related to the main findings.

3. The authors consider the fitness decrement associated with CD38- cancer cells to be fixed. However, it is likely that persisters under drug selection undergo further evolution and ultimately become fully drug resistant (i.e. they can grow quickly in the presence of drug). The authors could consider modeling this evolution from drug tolerance to drug resistance by adding one additional compartment with small per time step transition probability.

While we agree that adding such a compartment would model a very important biological mechanism, it is beyond the scope of the current analysis. This is because it would significantly complicate the optimisation problem. Instead of a simple tradeoff between the costs of drug treatment and cancer persistence, we would also be aiming to minimise the fully resistant population created in the process, either to stay within the level that can be controlled by the immune response, or to delay the emergence of resistant disease as long as possible. However, given the importance of the issue raised by the reviewer, we have added the following text to the discussion:

An interesting extension to our model would be to include a permanently resistant population which increases in response to treatment. Optimal drug treatment in the face of such acquired resistance is an important area of ongoing research.

4. The figure captions are too general and do not provide enough specific information about the information described to allow the figures to stand alone.

We have substantially improved the figure captions so that the figures can now stand alone.

5. The figures all share the same design, which makes it a bit confusing to parse out what is actually different from one figure to the next, particularly when combined with the point above.

We have expanded the captions and moved three figures (formerly Figures 5, 6, and 7) to a new supplementary materials section (Section S2.1). Together, these changes should alleviate this concern.

## Minor Comments:

1. The authors place their work in the context of other methods that apply optimal control theory to mathematical models of cancer evolution. However, they would also be served by discussing related work on mathematical models of microbial evolution (Weaver et al in PNAS as one recent example). As a disclosure, I was involved with that study – feel free to just mine the references for relevant literature. There have also been several recent model-free treatment optimization approaches that leverage RL (**Gallagher et al, 2024 among many others**).

These papers are certainly useful context, and we were happy to use the Weaver paper as it looks like a good entry point into this line of research. We couldn’t see how to include this topic in the introduction without undue distraction. However, we have incorporated the topic into the Discussion as follows.

Another critical context is microbial immunity; see for example [42], which optimizes antibiotic therapy using reinforcement learning, a machine learning method which has shown promise as an alternative to traditional optimal control theory. Reinforcement learning has also been used in recent research on cancer therapy [43].

2. A few more lines in the intro describing Multiple Myeloma (i.e. it’s prevalence, mortality rate, likelihood of evading treatment, cost estimate for treatment if available) may help set the stage a bit better for the rest of the study.

We have added this additional context to the Introduction (new text underlined):

The burden of morbidity and mortality is substantial, with an estimated 187,952 cases and 121,388 deaths globally in 2022, ranking 21^st^ and 17^th^ respectively amongst cancer sites [1]. A number of treatment options are available, although a complete cure has proved elusive [2], [3]. This often results in multiple lines of therapy being used in succession, leading to high costs. A 2022 United States study considered patients with refractory multiple myeloma after progressing through at least 4 lines of therapy, and found average total treatment costs of US$670,561 over an average 21.9 month follow-up period [4].

3. Figure 1 would be easier to digest if the notation was defined either in the figure or in the caption (i.e. what do A, P, and N) refer to in the different compartments.

We have added definitions to the caption. It now reads:

Figure 1: Model of multiple myeloma treatment with Daratumumab (control, *u*), including an immune response (purple arrows) and drug resistance mechanisms via loss of CD38 expression. The cell populations are healthy marrow cells (*A*), CD38+ cancer (*P*) and CD38- cancer (*N*), and all arrows represent movement of cells between populations or states. Three drug actions are included (green arrows): cell mortality and loss of CD38 expression in the CD38+ cancer cells, and off-target cell mortality in healthy cells within the compartment.

4. Related to above – more explicitly labeling the states in your results figures would be nice as readers won’t need to reference figure 1 or the methods to see what the notation means.

We have expanded the figure captions to include parameter meanings as well as noting major trends.

5. What does state refer to explicitly in your results figures? My brain assumes it is the proportion of the population that resides in each state but that doesn’t make sense because they don’t add up to one at any point.

State refers to proportions of the theoretical carrying capacity. We note this prior to the model ODEs (Equations 4, 5, and 6), saying:

The complete model (Fig. 1) is as follows, where the three state variables **x** = (*A*, *P*, *N*) are each expressed as a proportion of the marrow carrying capacity.

We have added this information to the caption for Figure 2 also, the first place these values are plotted.

6. Explicitly stating what alpha refers to somewhere in figure 4 would be a nice quality of life addition.

The caption to Figure 4 now reads:

Figure 4: Optimal control solutions for a range of values of *α*, which controls the strength of the immune response; default value is *α* = 0015. Drug duration is reduced for both cost functions as immunity is strengthened (left to right).

7. The authors should consider using more data rich representations for their sensitivity analyses – defining some summary statistic for control and then showing a heatmap for wide range of parameter values would give us a more deeper understanding of parameter regimes than the plotted trajectories.

This is an interesting idea. However, we were unable to identify any specific pair of model parameters that were of sufficient joint interest in the context of our other results to justify this bivariate analysis.

8. Tense isn’t consistent in the discussion (past or present tense are used interchangeably.)

We now consistently use past tense in the description of the results and the inferences drawn from them, which is most usage. Present tense is used for the mechanisms of the model, cost function etc. (e.g. “myeloma cells resist the effect of Dara”), as these are treated as existing outside time. Present tense was also used in a few cases such as for fresh conclusions (e.g. “we conclude that these controls are of less interest than the linear cost controls,”).

# Reviewer 3

Review of "Optimal control of Multiple Myeloma assuming drug evasion and off-target effects" by Lefevre et al.

The authors propose an ODE-based model for Multiple Myeloma (MM) plasma cell cancer treated by a monoclonal antibody medication (Daratumumab), which targets the CD38 receptor usually over-expressed in myeloma cells.

The two main complications are

* that the CD38 expression might switch off in (some of) the cancer cells (resulting in a cancer subpopulation unaffected by the drug) and

* that many healthy cells also have a significant level of CD38 expression (resulting in negative side-effects for the patient).

The model also assumes a (constant rate) influx of upstream healthy-cells, a shared carrying capacity for the competing healthy and cancer cells, and an immune response (modeled by Michaelis-Menten terms in the ODEs).

The authors then apply the tools of control theory (via Pontryagin Maximum Principle, PMP) to find the "optimal" rate of administering Daratumumab. The optimality is defined over a fixed time horizon and with respect to two different optimization criteria. A substantial part of the paper is devoted to analyzing the simulation results based on these drug/control policies and comparing their qualitative behavior in response to parameter variations. The paper is concluded by the Methods section, which provides a fairly detailed description of numerical methods and a stability analysis of no-drugs equilibria.

As an applied mathematician working in the same space, I value papers that advance the use of optimal control techniques in cancer modeling. However, I have significant concerns/questions (detailed below) about some of the fundamental modeling and optimization choices/assumptions made by the authors. I am also not sure whether the current style of the narrative is a good match for the majority of PLOS CB target audience. I thus recommend major revisions, expecting that the resubmitted version will be substantially different.

## Major Concerns/Comments:

=========================

### 1) The style/logic of the narrative and the scarcity of biological details

The manuscript is mostly well-written, with a significant amount of details included to explain the mathematical model and the drug policy optimization based on it. It is also great that the authors are candidly discussing the computational challenges (e.g., associated with approximating optimal bang-singular controls), but the logic of the narrative appears somewhat strange: the authors occasionally make it sound like some of the modeling/optimization choices are made purely for mathematical & computational reasons. They often do not provide sufficient information for readers to evaluate whether those modeling choices/assumptions are biologically justifiable.

In general, the paper is very light on biological details. E.g., there is a separate section 1.1 on dynamical systems & optimal control, which is written in a very general way. It includes 2 paragraphs on some ODE-based cancer models, but provides no information on why prior models are not suitable to describe MM and Dara-based therapies.

After all, cancer modeling literature is vast and the emergence of drug-resistance + damage due to drug toxicity are prominent feature in many papers -- so, what is different here? Why is the proposed new model an improvement?

Explaining this is particularly important since the model is at the heart of the paper while the control techniques employed by the authors are mostly fairly standard.

We have made numerous changes and additions to the Introduction and Discussion, as well as performing additional analyses, which we believe should alleviate these concerns.

To the best of our knowledge (and in the expressed opinion of Reviewer 1), incorporating resistance via reversible loss of expression and off-target killing of healthy cells in a single model is novel. In addition, the cyclic control form is unique in this class of models. As such, we believe this work is a significant contribution despite its theoretical nature and lack of calibration to real data. We have added text at several points to help clarify these contributions and limitations. For example, we added the following text in the Discussion:

Our study is largely theoretical, using parameters that have not been quantitatively fit to data. Indeed, mechanisms such as those in [13] are unquantified; the natural variation of CD38 expression between myeloma cells, and the rate at which this changes naturally and in the presence of Dara, is unknown. As a result, our optimal control results do not serve as clinical recommendations. Rather, they highlight how the different aspects of the disease are expected to influence how the disease best be treated.

We have worked to improve the biological context and justifications throughout the manuscript; we list some examples of changes below.

In the Introduction we have added background on myeloma and the use of Dara, and in the Discussion we briefly consider our results in the context of real treatment constraints:

In clinical practice, Dara treatment consists of discrete multi-hour infusions, typically repeated at weekly or longer intervals, with multiple dosing levels used [40]. Any attempt to calibrate our model to real data and draw a closer connection to clinical practice would have to account for this intrinsically intermittent process (as well as pharmacokinetics); optimal controls containing extended periods of low dose treatment would need to be interpreted in this context.

When we introduce the model in Section 1.2, we expand the justification of the core mechanism and acknowledgement of the simplifications made:

The drug resistance mechanism requires that cancer cell CD38 expression can vary, and the Darwinian selection mechanism requires natural variation in the population. We do not know the specifics of this distribution, so we assumed a simplified binary model.

We address the assumptions involving CD38 expression switching with a supplementary analysis (Section S2.2), and provide some support for the assumption that loss of CD38 expression reduces both proliferation and survival in myeloma:

It has been shown that CD38 is a growth and survival factor in a lung cancer model system [5], and so it is reasonable to assume that *N* has both lower proliferation and higher death rate than *P*.

We provide additional explanation for our cost function choices in Section 1.3, noting the role of tractability in the traditional popularity of the quadratic cost function:

In the absence of specific information, a simple linear combination of the cost factors is the most natural choice for the cost function. Quadratic functions are also widely used, with the justification that super-linear costs are evident in many contexts. This may also be due in large part to their superior mathematical and numerical tractability compared with linear functions [32].

We also include a sensitivity analysis for the cost function weighting assumptions (Section S2.3), and in the Discussion we now note the greater support for linear costs in the literature:

Indeed, the literature provides little support for a super-linear health burden of either cancer [37] or Dara [38, 39].

### 2) Modeling assumptions

Since the presented model is meant for a theoretical study only, it would be unreasonable to ask for a detailed justification of all biological mechanisms. But I think the readers would still expect to learn why specific assumptions are reasonable (even if clear simplifications). Here are a few questions I was left with:

*** Why is there a shared carrying capacity for A, N, and P cells?**

Does it represent some competition for resources or volume constraints?

If the latter, is it really compatible with a constant influx of healthy cells assumption?

Note that even in the absence of N & P, the number of A cells will equilibrate above 1.

Spatial crowding is a factor in multiple myeloma, as noted in the introduction:

“Myeloma can crowd out healthy marrow tissue,…”

The carrying capacity was adopted from the Crowell *et al.* model, where progenitor cells and cancerous cells compete for the same resources/space, but haematopoietic stem cells reside in a distinct niche [30]. Within that niche, if stem cells are in equilibrium there will be a fixed level of progenitor production due to stem cells, and the model indeed assumes that all of these progenitors become part of the population outside of the niche. This results in a constant supply term that is unaffected by carrying capacity, and the reviewer raises a good point about the implications of this. We do not expect incorporating rate limitation on the supply from stem cells would significantly affect the results we present, given that carrying capacity is not reached in practice in our results and healthy cell influx is only a quarter of the rate of healthy cell reproduction. However, we now acknowledge this point in the Discussion:

Firstly, we emphasize that our modelling effort is quite theoretical, focused on a particular mechanism of drug resistance and its consequences for optimal drug treatment. Although we believe that factors such as a space constraint and immune response are relevant to multiple myeloma, the mechanisms adopted from the Crowell model [30] are not adapted to the specific biology of multiple myeloma. The core drug resistance mechanisms were also simplified and lack calibration to real data.

*** Why is it reasonable to assume that the immune response is independent of the current A abundance?**

We acknowledge that the immune response in the model is a great simplification of the true biology, as we now note in the Discussion text quoted above. In Section 1.1, we say (new text underlined):

In this work we take a different approach, incorporating a very simplified immune response…

Immune proportionality to *A* would introduce a feedback mechanism to the system that would be directionally similar to the space competition. Since the variation we saw in *A* was relatively smaller than that in *C* = 1 – *A* – *P* – *N*, we expect that such a feedback mechanism would not substantially affect the control. Hence, although immune proportionality to *A* is biologically plausible, we have not pursued this mechanism. We note that our model does capture key aspects of the immune response, namely that cancerous cells trigger an immune response and the body has a maximal capacity of immune response (saturation of Mickaelis-Menton).

*** The following paper develops a much more detailed model of the immune response to MM: https://www.science.smith.edu/~ntania/papers/Gallaher_Springer_2018a.pdf**

Is it compatible with the authors' basic assumptions?

The cited paper is primarily concerned with modelling the very complex immune response, which is not the focus of our work. To emphasise this point, the final paragraph of Section 1.1 now says (new text underlined):

In this work we take a different approach, incorporating a very simplified immune response and placing focus instead on the drug resistance mechanism and off-target effects discussed above. See [29] for a detailed mathematical treatment of immune dynamics in multiple myeloma.

*** In many Figures (starting from 1c,d,g,h and throughout), the computational experiments start from a (drug-free) steady state with the highest observed abundance of cancer cells.**

Does such equilibrium actually exist for MM?

Is this a reasonable initial condition for treatment?

Wouldn't a patient die long before this equilibrium level is approached?

This is a simplifying assumption: we have added the following explanation and acknowledgment to the Discussion:

The use of a stable equilibrium as the initial state, adopted from [31], provided a consistent basis for comparison of optimal controls, and can be regarded as a theoretical worse case in terms of the difficulty of gaining control. A more realistic initial state would be based on a selected stage in a calibrated model of cancer progression.

*** Many of the tests seem to imply that, at least for a range of parameter values, the system is bi-stable, with a cancer-free equilibrium (A_0, 0, 0) also stable, and the natural (drug-free) dynamics leading to it once P is sufficiently suppressed by drugs.**

Was this bi-stability confirmed at least numerically for the case alpha>0 ?

Did the authors try to identify the separatrix for the basins of attraction?

Is this bi-stability biologically justifiable?

Will the real MM population continue declining to zero _after_ the drug therapy is finished?

The bi-stability is confirmed numerically. As described in Section S1.5 (Supplementary Methods / Equilibria), stable equilibria with *N*, *P* > 0 are found by applying Runge-Kutta to convergence, using the $\alpha=0$ equilibria as initial values. We can check stability of the *N* = *P* = 0 solution analytically, even when $\alpha>0$. We have added a substantial supplementary analysis (Section S2.4, Extended and infinite horizon validation) which includes the following explanation that we confirmed that the *N* = *P* = 0 state is stable when this is shown as the endpoint in the optimal control solutions:

In all cases with $\alpha>0$ the computed optimal control shows the control and cancer level at or approaching zero by the end of the time window, implying no ongoing costs. In each case this was confirmed by an infinite horizon minimum cost solution u = N = P, confirming that no delayed recurrence would occur under our model.

We have numerically mapped the basins of attraction for the default model over the [0,1]^3 cube with 0.1 resolution, supporting the assumption of two stable solutions (it is easily seen that no physical equilibria can exist outside this region). Given the 3d state space, we could not identify a method of numerically mapping the separatrix and displaying this information in an informative and concise form, but are willing to implement a suitable suggestion, at editorial discretion. For now, we have made a note of this check in Section S1.5 (Equilibria):

We cannot prove uniqueness for this steady state, however we performed a numerical check for the default model parameters. We used a grid search with initial values *A*, *P*, *N* ∈ {0, 0.1, 0.2, …, 1}, calculating the trajectories and determining that every trajectory converged towards either (*A*, *P*, *N*) = (0.318, 0.381, 0.015) or (*A*, *P*, *N*)=(0.423, 0, 0) (it is easily seen that no physical equilibria can exist for *A*, *P* or *N* greater than 1, except possibly when *N* = *P* = 0).

As discussed in the previous response, this assumption is a simplification of the true biology.

*** The authors use the "general overexpression of CD38 in myeloma" to argue that proliferation rate rho is higher while the natural death rate mu is lower for P cells compared to N cells _and_ that the rate of P --> N transitions should be much lower than the rate of N --> P transitions.**

Is there any biological evidence for both statements to hold true?

(Either of these assumptions can produce experimentally observed overexpression of CD38. Why assume both?)

Fig.5 provides some experiments on the efficacy of optimal drug therapies when both delta_P and delta_N are scaled down proportionally, keeping their ratio fixed.

If there is no biological reason to believe that the ratio of 10 is reasonable? Wouldn't it be prudent to conduct some tests with smaller ratios? (In all included experiments, the abundance of N cells remains fairly low with or without drugs. Does this correspond to what is observed about MM in practice?)

We did not have data to inform this choice, and so considered that the most neutral assumption would be that the predominance of *P* over *N* was based on a combination of factors. The ratio of 10 to 1 was certainly arbitrary. Hence, we followed your suggestion and conducted sensitivity analyses (Section S2.2. Sensitivity analysis for expression switching ratio, also referenced in the model introduction):

The model was designed to ensure a predominance of *P* over *N* in the absence of a forcing mechanism such as the drug control. We assumed that this predominance resulted from a combination of lower fitness in *N* and a much higher propensity to gain than to lose expression (*δ_N_*/*δ_P_* =10). While we considered the effect of varying the fitness differential (Fig. S2), the *δ_N_*/*δ_P_* ratio was conserved even when we considered the effect of changing the general rate of expression switching (Figures 6, 7, 8, S1). Since this ratio is purely an assumption, we briefly test the effect of reducing the ratio. We fix each of the default values *δ_N_* = 0.03, *δ_P_* = 0.003 in turn, and reduce the ratio to 3 and then 1 (Fig. S4). Comparing with Fig. S2, we see that when the ratio is 3 there is minimal difference from the corresponding case with ratio 10 and the same value of N, suggesting that there is limited sensitivity to the specific value of *δ_N_*/*δ_P_* ratio provided that it is significantly greater than 1. More substantial differences are apparent when *δ_N_* = *δ_P_*, although we still produce bang-singular controls for the linear cost function, and see no major differences in control form. The initial value of *N* in these cases remains relatively low, implying that the fitness difference is sufficient to maintain the required predominance of *P* in the absence of the control. Therefore, a model with *δ_N_* = *δ_P_* is plausible, although we do not investigate further in this work.

Please address all of the above points in the paper -- your readers will be grateful.

### 3) Optimization: objectives, horizons, interpretations

*** It seems strange that the running cost includes no penalty for the depletion of healthy cells (A).**

The abundances of P & N are only a part of the story. The same "successful" therapy might be prohibitive if starting from a lower A abundance. If the authors don't want to do this, another alternative is to introduce a state constraint -- the minimum level of A allowed throughout the process.

If the authors decide against these changes, they should explain to readers why ignoring this aspect is reasonable.

In a sense, our running cost does include a penalty for the depletion of the healthy cell population, A. Both choices of cost function assign higher cost to higher levels of treatment, and higher levels of treatment result in greater amounts of healthy cell death. In a similar fashion, the linear-cost controls constrain the maximum treatment level, and hence constrain the extent of off-target cell death deemed reasonable. All of that said, we agree that a cost function whose formulation explicitly considered the level of healthy cells would be meaningful. The challenge is that it is unclear what exactly this formulation should be, or how it ought to be parameterized. For the purposes of our theoretical study, we remain limited to a generic cost for treatment, but in the modified manuscript we now explore how optimal treatment varies when the relative health costs assigned to cancer burden and treatment are changed.

In the updated version of the manuscript, we discuss this suggestion as an extension of interest, where the cost functions are introduced (Section 1.3):

Note that the control term of the cost function is intended to include the health impacts of drug toxicity. As our model includes the off-target effects of drug treatment, the control term is directly linked with the death of healthy cells and so captures health impacts in this sense, although its generic form permits broader interpretations that also incorporate other side-effects of drug treatment. A plausible alternative that we did not adopt is to explicitly describe the health impacts due to loss of healthy cells, for example by including an additional term based on the gap between A and an assumed healthy level.

*** The authors are considering a fixed-horizon optimal control problem with no terminal cost, which can creates all kinds of strange "the deadline is near" effects (as they correctly point out at the end of section 1.3).**

The authors handle this by assuming that the planning horizon is large (T=200) when computing optimal controls, but then using those controls and plotting all trajectories for a much shorter time interval (e.g., [0,T_1] with T_1 = 50 or 100). This is not a standard/robust approach since one never knows a priori whether the T/T_1 ratio is large enough. At the very least, it is necessary to recompute the optimal control for the horizon 2*T and check that on [0,T_1] it remains virtually unchanged. A much better / theoretically-sound approach is to use a suitable terminal cost $\phi(A,P,N)$, which should properly assign a higher penalty for N rather than P at the end of the process -- since a sizable CD38- population makes that cancer harder to control and the recurrence more likely.

The authors assertion that $\phi=0$ is "generally preferred as it provides more tractable computations" seems very strange. PMP is routinely used with nonzero terminal cost in a variety of applications.

* The authors are at times discussing a possibility that "permanent control" is needed and of finding an "optimal steady state treatment" for "indefinite maintenance" and of projecting "ongoing costs from the trendline". All this is not really compatible with the fixed-horizon formulation -- the authors indirectly acknowledge some of these difficulties (e.g.,on line 239), but their readers deserve an explicit and detailed discussion of what they have in mind.

A rigorous case for the "steady state treatment" would require a different optimal control formulation -- with infinite horizon and either time-discounting or time-averaged optimality.

The assertion that *ϕ* = 0 is "generally preferred as it provides more tractable computations" was poorly considered and has been removed.

We have not been able to formulate terminal costs which reasonably represent the costs of ongoing treatment; the long-term cost of a very small amount of residual cancer may vary dramatically depending on whether the cancer cell population will expand again without treatment, and what level of treatment is required to prevent or limit such a recurrence. Instead, we have added a supplementary analysis (Section S2.4) to address these issues.

Originally, the main justification that we used to extrapolate from the finite window to indefinite treatment regimes was to note convergence to a steady state which we could interpret as an indefinite treatment regime. Therefore, our primary supplementary analysis was to validate this assumption by directly calculating the infinite horizon constant optimal control for time averaged cost. Most often the apparent long-term state was just *u* = *P* = *N* = 0, which could be checked analytically by testing stability of the *P* = *N* = 0 equilibrium. Otherwise, a numerical method was used (detail in Section S2.4). The secondary analysis was, as suggested, recalculating several of the optimal controls on a doubled time window, and checking for agreement on [0,100]. The cases tested were selected for being representative of the most doubtful cases. These two analyses highlighted a few edge cases. Perhaps most importantly, it appears that the ongoing cyclic solution form will always be subject to a finite window effect on the timing of the intermittent treatment, although this diminishes as the ratio between the time window and the cyclic period gets larger.

The penultimate paragraph of Section 1.3 has been rewritten to reflect the points above, and to reference the supplementary analysis:

The cost function cannot take into account the system state outside the selected time window (for example, cancer recurrence). The function *ϕ*(**x**(*t_f_*)) allows us to assign a cost to cancer that remains at the end of the evaluation window, but we lack a reasonable *a-priori* estimate of the associated long term cost: for example, the cost associated with a small residual cancer population will depend strongly on whether it will rebound without treatment. For this reason we set *ϕ*(**x**(*t_f_*)) = 0, and instead use extended time windows and evaluate the infinite horizon optimum solutions to check whether each optimal control calculated is free of finite time window artefacts, and reaches either a stable cancer free state or an optimal maintainance treatment regime within the time window (Section S2.4).

The results section has been edited at several points based on the findings of this analysis. We have also added a substantial paragraph on methodology in the discussion, which includes the following:

The interpretation of results from a finite time window method in an intrinsically infinite-horizon context also demands careful consideration. In most cases we were able to demonstrate that the controls converged (in the time variable, not iterations) to an infinite-horizon, optimal time-averaged cost constant control; most often this was complete elimination of the cancer (Section S2.4). An exception was the indefinitely repeating bang-bang solution form, for which the window length appeared to bias the cycle timing. Aside from these two specific issues, our sensitivity analyses indicated that the control forms we presented are robust; for example, in Section S2.3 we saw that for a composite but mostly linear cost function, the control obtained using the continuous control algorithm approximated the bang-singular form.

*** It is not clear whether u and u^2 used in their running costs are meant to reflect the monetary cost of drugs only (which makes u^2 hard to justify) or also reflect the overall burden of treatment for the patient.**

The discussion on lines 367-377 seems to provide two contradictory answers to that question, additionally muddled by a sentence on lines 216-217 on p.11. It seems that a more logical choice would be to use a more general superlinear cost; e.g., $u + \kappa u^2$, where a positive parameter $\kappa$ could be tuned as needed.

Note that repeating the computation with a sequence of decreasing $\kappa$ values could produce a nice sanity check: the overall cost would tend to what can be found in the authors' discontinuous control optimization (with the running cost L=u+P+N).

While a more general cost function is likely to be more reflective of reality, we do not know what functional form would be most accurate. Hence, we have retained our general approach of considering both linear and quadratic costs. In addition, we have done a sensitivity analysis along the lines you suggested (Section S2.3: Cost function sensitivity analysis). We considered two weighted average functions, and the results indicated that: (1) an intermediate cost will give an intermediate control form; and (2) there is continuity between controls produced using the different algorithms (the sanity check you suggested). This supplementary analysis (which is grouped with an analysis of cost term weights suggested by Reviewer 1) is referenced in the cost function section of the introduction, along with some justification of our cost function approach:

Furthermore, a realistic super-linear cost function would likely resemble some unknown combination of linear and quadratic terms. Thus our sensitivity analysis also considers a general super-linear cost function containing both linear and quadratic terms, to investigate whether results derived from this ‘mixed’ cost function are broadly intermediate between those generated using purely linear or purely quadratic cost functions.

Lines 216-217 in the results have been reworded as follows:

However, if the true costs include any linear component such as the financial cost of drug supply, the quadratic cost function will be least accurate at lower levels of control and cancer. Hence the very low cost associated with an extended period of low-level treatment may not be realistic.

The discussion has been substantially revised based on these points and the related comments of Reviewer 1. The main discussion of this issue is now the following paragraph:

The quadratic cost function results helped to demonstrate the sensitivity of the control form to the cost assumptions, as well as the robustness of many of the trends we identified. However, we conclude that these controls are of less interest than the linear cost controls, notwithstanding the greater ease of computation. Indeed, the literature provides little support for a super-linear health burden of either cancer [37] or Dara [38, 39]. Even if super-linear costs do exist, the very low cost assigned to an extended period of low dose treatment is unlikely to be realistic; if real costs include at least some linear factors, such as the financial drug cost, these will tend to dominate as *u* and *P* + *N* approach zero. This unrealistic low cost gives rise to the tapering form of the quadratic cost controls, and appears to substantially obscure the effect of the drug resistance mechanism, particularly in terms of the prolongation of treatment. In contrast, for the linear cost function any period of ongoing control at a reduced level can be reasonably attributed to the biological mechanisms that we model.

*** For the first examined running cost (L=u+P+N), the authors are referring to the resulting control as "bang-bang" throughout the paper.**

But this leads to a significant terminological confusion: this term is uniformly used in the literature in situations where the optimal control takes extreme values only. Problems, in which the Hamiltonian is linear in u but the switching function \psi might stay equal zero over some interval, yield "singular control arcs" with $u \in (0,1)$ for that duration. Whenever singular arcs are present, the standard nomenclature is "bang-singular" or "bang-singular-bang" controls -- precisely the setting for many examples in the current paper (e.g., top right panel in Fig 3 and top row examples in many subsequent Figures).

The authors should switch to this standard terminology to avoid confusing their readers.

We thank the reviewer for this clarification of terminology (see also similar points from Reviewer 1).

We now refer to the two cost functions (rather than incorrectly using the terms “bang-bang” and “continuous” control). We sometimes use “bang-bang” and “bang-singular” to describe the control forms.

In Section 2.2, after presenting the optimal controls for the default model, we have added the following text:

Linear costs typically result in optimal control levels at either the maximum level or zero. In the Null-N model all solutions consisted of an initial period at maximum level followed by an abrupt and final end of treatment. This is known as a bang-bang control, although this term may refer to controls that are explicitly constrained to either a maximum or minimal value at each time ([34, 35], or more recent work such as [36]). In our model, however, the linear cost function produces an initial period at maximum level followed by a period of intermediate level control. This intermediate period is known as a singular arc, and the control is a concatenation of bang and singular controls which we refer to as bang-singular [32].

### 4) Optimization: numerical methods

*** It is well-known that PMP is merely a necessary condition for a local optimality of control/trajectory pair.**

[This is in contrast to the Hamilton-Jacobi-Bellman dynamic programming approach, which guarantees the global optimality, but at a higher computational cost of solving a PDE.]

Without special properties of a control problem, one cannot guarantee more tan the local optimality of a PMP-based solution. (And even this requires verifying a second-order Legendre-Clebsch condition.)

In general, a solution to the two-point BVP specified by the authors' equations (1,2,9,10) may not be unique.

Which among them will be recovered by a numerical method depends on the chosen initial guess for the control u. Only one among those solutions will correspond to a globally optimal control/trajectory and finding the right initial guess to converge to that global optimum can be quite non-trivial.

The authors should

- either explain why they believe this two-point BVP has a unique solution for their specific control problem

- or explicitly acknowledge that the obtained controls might be only locally optimal and there might well exist another control that produces a lower overall cost.

We now acknowledge this limitation in the introduction:

Our approach is based on Pontryagin's maximum principle [26]. We note that this approach does not guarantee a global optimum.

And in the discussion we say:

Finally, for either cost function the method used does not exclude the risk that the optimal controls are only locally optimal, and the result could in theory depend on our initial control choice *u*(*t*) = 0 for all *t*. The generally intuitive and explainable shape of the controls suggests that this risk was minimal; for example, an initial delay in treatment would not provide a benefit. Nevertheless, we must acknowledge this limitation.

*** The authors' approach to computing bang-singular controls is somewhat ad-hoc and the resulting controls likely contain numerical artifacts.**

All of their graphs with bang-singular controls show an overshoot -- a brief sharp dip in the u value whenever the singular arc starts, followed by a brief (much smaller) increase and a slower, seemingly linear-in-time decrease in u. That initial downward overshoot looks strange. The authors should check whether it remains unchanged or decreases/vanishes under numerical refinement -- i.e., when recomputed with much smaller time-steps in their Runge-Kutta solver and with more stringent convergence criteria in their forward-backward iterations.

If these overshoots are indeed numerical artifacts, this should be acknowledged explicitly in the paper.

It is also worth mentioning that bang-singular controls are an active research area and that there are a few well-developed numerical methods for computing them accurately. See, for example,

https://doi.org/10.1007/s10589-022-00350-6

https://doi.org/10.1016/j.compchemeng.2020.106923

and references therein. The authors might find it worthwhile to switch to one of these instead.

We had initially thought that this was probably a degenerate form of the break between the bang and singular arc sections observed in some controls, but after careful examination we agree it is most likely an artefact. Increased Runge-Kutta resolution made no difference to this overshoot, but greatly extended iterations of the algorithm led to a very slow contraction of this feature (horizontal reduction but little or no reduction in vertical magnitude; in contrast the break in a bang-zero-singular control was stable). We prepared a figure showing the effects described above, but decided that it did not warrant an additional supplementary section. Instead, we briefly note this finding when we acknowledge this artefact in the last paragraph of Section where it is first seen:

The bang-singular control solution includes a small oscillation at the start of the singular arc, going briefly below and then above trend. This feature is invariant under increased numerical resolution, but additional iterations of the algorithm resulted in a very slow contraction of the oscillation in tandem with gradual steepening of the vertical drop immediately prior. We conclude that the oscillation is a local numerical artefact associated with imperfect convergence to large step changes in the control, and should be discounted. This does not apply to the general form of the control, or to the more substantial breaks in control identified in cases such as Fig. 6.

This artefact seems to be linked to the large drop in control immediately prior, and the very slow convergence towards a true vertical representation of this discontinuity. Convergence is also slower in general for controls with multiple discontinuities. We conclude that this is most likely a fundamental limitation of using a single convergence algorithm to obtain a bang-singular control, suggesting that a more complex composite approach (as in the work you suggested) is needed to efficiently find these solutions. However, after considering your points and undertaking additional analysis we believe the optimal controls we obtained are robust except for this artefact and the finite window timing bias in the ongoing cyclic solution. Hence, we decided it was unnecessary to try to recalculate the results with more advanced methods, and have instead acknowledged the issues and added some discussion (including the references you provided).

… this convergence was imperfect at discontinuities, with local artefacts at bang-singular interfaces … Aside from these two specific issues, our sensitivity analyses indicated that the control forms we presented are robust; for example, in Supplementary Section S2.3 we saw that for a composite but mostly linear cost function, the control obtained using the continuous control algorithm approximated the bang-singular form. However, the calculation of bang-singular controls is a subject of current research (for example [44, 45]), and the artefacts we saw point to the value of these more complex, multi-stage methods in order to efficiently find these controls with full fidelity.

## Minor comments:

* the word "on" on line 54 (p.3.) should probably be deleted;

We have removed this word.

* the statement on lines 101-102 (p.5), just after formula (3), is only correct when f is also linear in u.

This is true for your example, but at this point in the paper you are still discussing a general control problem with a general f.

We now say:

If ℒ and f both have a linear dependency on u, then bounds must be imposed on u in order to give a well-defined solution.

* line 124, p.6: "cancer calls" --> "cancer cells"

We have corrected this typo.

* p.7 It would be really helpful to provide some information on the hypothesized mechanisms of expression switching. This will make it much easier to evaluate the suitability of terms in the ODE and the realistic range of values for the relevant parameters.

We know that the expression decreases after Dara treatment and can recover in time, and a natural range of expression across the population with some level of random walk behavior within this range seemed the most likely method of recovery. Without knowing the actual variation of expression, we assumed a binary system as modelled. We added the following lead sentences to the explanation of this part of the model in Section 1.2:

The drug resistance mechanism requires that cancer cell CD38 expression can vary, and the Darwinian selection mechanism requires natural variation in the population. We do not know the specifics of this distribution, so we assume a simplified binary model.

* line 140: please define "fitness"

We have added the following sentence after the referenced statement to explain and justify the assumption:

It has been shown that CD38 is a growth and survival factor in a lung cancer model system [5], and so it is reasonable to assume that *N* has both lower proliferation and higher death rate than *P*.

* p.8: The paper states that "Where possible, parameter values were adapted from the Sharp model, which were selected to produce balanced dynamics supporting both healthy and cancerous states and the capacity for effective drug control."

Please provide a precise statement on which parameters have exactly the same values as in the Sharp model.

We have added an extra column for the Null-N model parameters in the table, and added the following explanation of parameter providence (new text underlined):

A description of the model parameters, and the default values used, are listed in Table 1. In the column on the right hand side we list a second set of parameter values which, together with the condition *N*(0), define the *Null-N* model. This simplification reproduces the core features of the Sharp model and is used as a negative control.

The non-zero parameters of the Null-N model were adapted from the Sharp model (Table 1 of [31]), which were selected to produce balanced dynamics supporting both healthy and cancerous states and the capacity for effective drug control. Our parameters *µ_A_*, *ρ_P_* and *µ_P_* correspond to *δ_A_*, *ρ_L_* and *δ_L_* respectively in the Sharp model, *β_A_* = *δ_S_*(1 – *δ_S_*/*ρ_S_*) is the exit rate from the *S* compartment at steady state, and the remaining parameters retain the same name. These values are also used in the full model, except for a small adjustment to *ρ_P_* and *µ_P_* to balance the introduction of *N* with substantially lower proliferation and higher mortality. The additional parameters in the full model were described previously, other than the Dara related parameters which we consider now.

* line 155: "population N substantially less fit" --> "population N is substantially less fit"

We have added this missing word.

* p.10 & beyond: Please introduce the "Null-N model" formally.

Please don't refer to it as "Sharp model" in figure captions.

The Null-N model is now specified in the introduction, with parameter values listed in the table alongside the full model. This arrangement is used to help better explain the parameter derivations, as requested above. The Null-N name is used in figure captions and elsewhere.

* line 189 states that $\mu_u = 0$.

I suspect that you meant $\mu_{Au} = 0$.

We did mean that, although this text has been removed since the definition of the Null-N model has been moved to the introduction and integrated with the table of parameters, as noted above.

* Section 2.2: please specify explicitly what is meant by a "prolonged treatment".

We have modified the text in this section, including the following clarification (new text underlined):

In both cases, the total control and overall cost is increased relative to the Null-N control, and the duration of treatment is extended; the prolongation of treatment is less clear for the quadratic cost control due to its tapering form, but note that a smaller proportion of the total control is applied in the first 5 or 20 time units for the full model.

* Fig. 3 and beyond: the legends are confusing.

E.g., I suspect that when you write $U^2 (t<5)$, what is really meant is $\int_0^5 u^2(t) dt$, but I am not completely sure.

Please explain this explicitly in the text. The same issue is also relevant in subsequent figures (e.g., bottom right Fig.4).

We have added this additional explanation to the figure 3 legend:

Each of these totals represents the integral of the respective function over the interval$t\in[0,T]$, where *T* = 50 unless specified otherwise.

We have added this similar explanation to the (b) legend of figure 4 and similar figures.

Each value plotted is the integral of the specified function over the interval Time $\in[0,T]$, where *T* = 100 unless otherwise specified. For the “Initial *U*” value, *T* is the maximum value for which *U* = 1 over the interval $[0,T]$.

* line 230: please define what is meant by the "final control of cancer".

We have reworded the sentence for clarity. It now reads:

As the immune response approaches zero, there is a point at which immune suppression becomes impossible at any cancer level, and the optimal control transitions to an initial high dose treatment followed by an indefinite maintenance treatment.

* line 245: "asyptotically" --> asymptotically"

We have corrected this error.

* Most of the experiments in section 2 can be viewed as sanity check; i.e.,

they don't produce insights beyond what one would expect without computing optimal controls.

(E.g., yes, more drugs will be needed to control MM if the immune mechanism is less effective or if drug-escapement is enabled. This seems quite natural.) I would recommend devoting more space to discuss a few unobvious observations -- e.g., the non-monotonicity of the overall cost as a function of drug-induced escapement under "continuous optimal" control -- the bottom right in Fig.7.

We believe that this issue has been alleviated by moving Section 2.4 into the supplementary materials.

* Section 2.6 is also interesting (might be worth drawing a connection to "metronomic" drug therapies), but the chosen fixed-horizon optimization framework makes it harder to discuss these results rigorously.

Definitely worth checking if these approximately optimal policies change much under numerical refinement.

The added supplementary analysis “Extended and infinite horizon validation” identifies a finite time window effect on the timing of the ongoing cyclic/metronomic control, and we note this caveat in the results and discussion. However, the forms of these controls are otherwise conserved when we increase the time interval.

* While it's up to the authors to determine the structure/composition of their paper, I did not find the "Conclusions" contributed much beyond what was already covered just above. I would suggest omitting section 3.1 altogether.

We agree that it didn’t really add anything, and we have removed this subsection.

* The usual format in PLOS CB seems to indicate that section 4 would look more natural as Supplementary Materials.

We have included this change in the restructure. See also reviewer 1 comments under significant revisions. The methods and the original results Section 2.4 have been moved into a supplementary section which also contains various additional analysis as requested.

* The discussion on lines 475-483 is hard to parse due to the choice of notation. I would advise against using the symbol $u'(t)$. Please also define formally what is meant by $u_t$.

We have changed this notation to *u**(*t*). We could not find any use of *u_t_* in the methods, but have added short definitions of *u*_old_ and *u*_new_.

* The authors provide a github repository link in the paper, but it appears to be invalid:

<https://github.com/jameslefevre/optimal-control/tree/main>

The corrected link is

https://github.com/jameslefevre/dara-optimal-control/tree/main

# Additional

Have the authors made all data and (if applicable) computational code underlying the findings in their manuscript fully available?

The PLOS Data policy requires authors to make all data and code underlying the findings described in their manuscript fully available without restriction, with rare exception (please refer to the Data Availability Statement in the manuscript PDF file). The data and code should be provided as part of the manuscript or its supporting information, or deposited to a public repository. For example, in addition to summary statistics, the data points behind means, medians and variance measures should be available. If there are restrictions on publicly sharing data or code —e.g. participant privacy or use of data from a third party—those must be specified.

Reviewer #1: Yes

Reviewer #2: Yes

Reviewer #3: No: The authors provide a github repository link in the paper, but it appears to be invalid:

https://github.com/jameslefevre/optimal-control/tree/main

PLOS authors have the option to publish the peer review history of their article (what does this mean?). If published, this will include your full peer review and any attached files.

If you choose “no”, your identity will remain anonymous but your review may still be made public.

Do you want your identity to be public for this peer review? For information about this choice, including consent withdrawal, please see our Privacy Policy.

Reviewer #1: No

Reviewer #2: Yes: Davis T Weaver

Reviewer #3: Yes: Alexander Vladimirsky

Figure Files:

While revising your submission, please upload your figure files to the Preflight Analysis and Conversion Engine (PACE) digital diagnostic tool, https://pacev2.apexcovantage.com. PACE helps ensure that figures meet PLOS requirements. To use PACE, you must first register as a user. Then, login and navigate to the UPLOAD tab, where you will find detailed instructions on how to use the tool. If you encounter any issues or have any questions when using PACE, please email us at figures@plos.org.

Data Requirements:

Please note that, as a condition of publication, PLOS' data policy requires that you make available all data used to draw the conclusions outlined in your manuscript. Data must be deposited in an appropriate repository, included within the body of the manuscript, or uploaded as supporting information. This includes all numerical values that were used to generate graphs, histograms etc.. For an example in PLOS Biology see here: http://www.plosbiology.org/article/info%3Adoi%2F10.1371%2Fjournal.pbio.1001908#s5.

Reproducibility:

To enhance the reproducibility of your results, we recommend that you deposit your laboratory protocols in protocols.io, where a protocol can be assigned its own identifier (DOI) such that it can be cited independently in the future. Additionally, PLOS ONE offers an option to publish peer-reviewed clinical study protocols. Read more information on sharing protocols at https://plos.org/protocols?utm_medium=editorial-email&utm_source=authorletters&utm_campaign=protocols

In compliance with data protection regulations, you may request that we remove your personal registration details at any time. (Remove my information/details). Please contact the publication office if you have any questions.
